# Biomedical Materials and Fabrication Methods for Construction of In Vitro Neurovascular Unit Models

**DOI:** 10.3390/ma19173590

**Published:** 2026-08-24

**Authors:** Yuanyuan Xu, Wenlong Yu, Yang Li, Lei Zhang

**Affiliations:** 1 Department of Mechanical Engineering, Tsinghua University, Beijing 100084, China; xuyuanxoo@163.com; 2 School of Mechanics and Safety Engineering, Zhengzhou University, Zhengzhou 450001, China

**Keywords:** biomaterials, blood–brain barrier, neurovascular unit, organ-on-a-chip, multifunctional printing, 3D printing, brain tumors

## Abstract

In vitro neurovascular unit (NVU) models are essential for reproducing blood–brain barrier (BBB) transport and neurovascular cell interactions. However, the literature remains fragmented: biomaterial chemistry, fabrication parameters and organ-on-a-chip architecture are commonly evaluated in isolation, while inconsistent reporting of matrix properties, processing history, cell source, flow and barrier readouts prevents head-to-head comparison and the extraction of transferable design rules. To address this gap, this review integrates biomaterials, manufacturing technologies and organ-on-a-chip engineering within a unified material–process–structure–function framework. We translate endothelial junctions, basement-membrane components and perivascular cells into experimentally actionable material requirements; compare natural, synthetic, semisynthetic and decellularized extracellular-matrix hydrogels; and examine crosslinking, peptide functionalization, stimuli responsiveness, composite-network formation and preparation methods. Findings from Transwell, microfluidic, tubular, self-assembled and 3D-bioprinted BBB systems are used to relate matrix stiffness, degradability, ligand density, permeability, device-body material and fabrication route to barrier maturation, analytical access and reproducibility. By defining matched controls and minimum reporting requirements for chemistry, mechanics, transport and processing, this review provides a practical basis for next-generation BBB models that can improve permeability and efficacy screening in drug discovery, reproduce disease- and patient-specific barrier dysfunction, and support individualized response testing with iPSC- or patient-derived cells.

## 1. Introduction

The blood–brain barrier (BBB) maintains the homeostasis of the central nervous system (CNS) by forming a tightly regulated neurovascular unit (NVU) [1,2]. Formed by the BBB and neural cells, the neurovascular unit serves as the fundamental structural and functional unit of the brain. As a protective barrier for the brain, the BBB features selective permeability to substances. Meanwhile, it impedes drugs from entering the central nervous system, hindering the treatment of neurological disorders such as psychiatric illnesses, cerebral infections, brain tumors, and neurodegenerative diseases. The progression of brain tumors triggers structural abnormalities in the BBB, leading to the formation of the blood–tumor barrier (BTB). Although the permeability of this barrier is elevated, it still exhibits prominent selective permeability to drugs, restricting drug accumulation within brain tumor lesions [3,4]. Preclinical research on BBB structure and function has traditionally relied heavily on animal models [5,6,7]. Nevertheless, drawbacks including low throughput and interspecies differences of animal models hinder the translational application of drug research to humans [8], constituting a rate-limiting step in drug development. Research focused on constructing in vitro BBB models aims to fully recapitulate the sophisticated and complex in vivo architecture as well as diverse physiological functions of the BBB, acting as a vital complement to animal BBB models. Accordingly, fabricating biomimetic BBB models with native-like structure and functionality is of great significance for thoroughly elucidating the microphysiological characteristics of the human brain and developing therapeutic agents for brain tumors.

Organ-on-a-chip systems combine microfluidic control with engineered extracellular matrices to expose cells to defined geometry, perfusion and biochemical gradients. Representative studies demonstrate the breadth of this approach: Maoz et al. linked endothelial and neuronal compartments to resolve neurovascular metabolic coupling [9], Kim et al. modeled fungal infection in a human NVU chip [10], and Ahn et al. used a microengineered BBB platform to study nanoparticle transport [11]. However, these studies also reveal a central literature gap: cell source, channel architecture, membrane or hydrogel chemistry, flow conditions and barrier readouts were changed simultaneously, so the contribution of the biomaterial cannot be isolated and results cannot be compared head-to-head. The 2025 meta-analysis by Shamul et al. confirmed substantial heterogeneity in model composition and reported properties [12]. Additional limitations include long fabrication and maturation times, low throughput, chip-to-chip and cell-batch variability, nonphysiological membrane thickness and stiffness, hydrophobic-drug sorption and oligomer leaching from PDMS, bubble formation, evaporation, leakage or delamination, limited access for TEER and abluminal sampling, incomplete immune and neuronal representation, inconsistent reporting of matrix mechanics and permeability, and difficulty scaling fabrication while preserving surface chemistry [13,14]. Thus, rapid prototyping alone does not establish physiological or translational validity [15,16,17].

Three-dimensional (3D) printing and bioprinting expand this material-design space by patterning cell-laden hydrogels, sacrificial templates and rigid device components from digital models [18,19,20,21]. Lee et al. printed perfusable vascular channels using gelatin liquefaction and collagen crosslinking [22]; Wang et al. produced a membrane-separated BBB chip with stereolithographically fabricated components [23]; and a recent coaxial-bioprinting study generated a layered vascular structure with BBB function for neuroprotective-drug screening [24]. These examples support the claimed advantages in geometric control and multi-material placement, but they also expose unresolved gaps. Cross-study comparison is confounded because nozzle diameter, extrusion pressure, print speed, light dose, photoinitiator, bioink concentration, crosslinking sequence and cell source are rarely matched [18]. Extrusion can impose damaging shear and has limited resolution; light-based methods introduce photoinitiator and radical toxicity as well as restricted light penetration; increasing polymer concentration improves shape fidelity but can reduce cell migration and solute transport; sacrificial inks may be incompletely removed; and multi-material constructs can delaminate or swell differentially [19,25,26]. Further disadvantages include slow printing, expensive equipment, sterility and batch-control challenges, printer-dependent reproducibility, limited vascular dimensions, and insufficient long-term validation of TEER, permeability and transporter activity. Accordingly, future BBB printing studies must compare materials under matched processing conditions rather than attribute improved function to printing alone [18,19,27].

This review therefore focuses on biomaterial selection and preparation for stable in vitro BBB and NVU models. Section 2 condenses BBB anatomy into experimentally actionable material requirements and identifies gaps in current models. Section 3 analyzes hydrogel chemistry, peptide functionalization, stimuli-responsive systems and composite networks. Section 4 compares the material basis of spheroid, Transwell, microfluidic and printed formats, whereas Section 5 distills design principles and synthesis or fabrication methods. Bioprinted organ-on-a-chip devices are then discussed as an emerging integration route rather than as the sole organizing theme.

## 2. Material-Relevant Architecture and Research Gaps of the Blood–Brain Barrier

For a biomaterials review, BBB anatomy is useful only when it changes material selection, fabrication or interpretation. Table 1 therefore serves as a location-to-material map rather than a general anatomical catalog: it lists the cells [28] and molecular structures discussed below [29,30,31], their native position [32,33,34,35], the material variable used to reproduce that position [28], recent experimental results and the corresponding readout [36,37,38]. This organization distinguishes a luminal vascular surface, a thin basement-membrane interface and a softer perivascular compartment, and it makes explicit which biological features are deliberately omitted from a given model [39,40,41].

### 2.1. Material-Defined BBB/NVU Interfaces: Recent Comparative Studies

**Figure 1:**
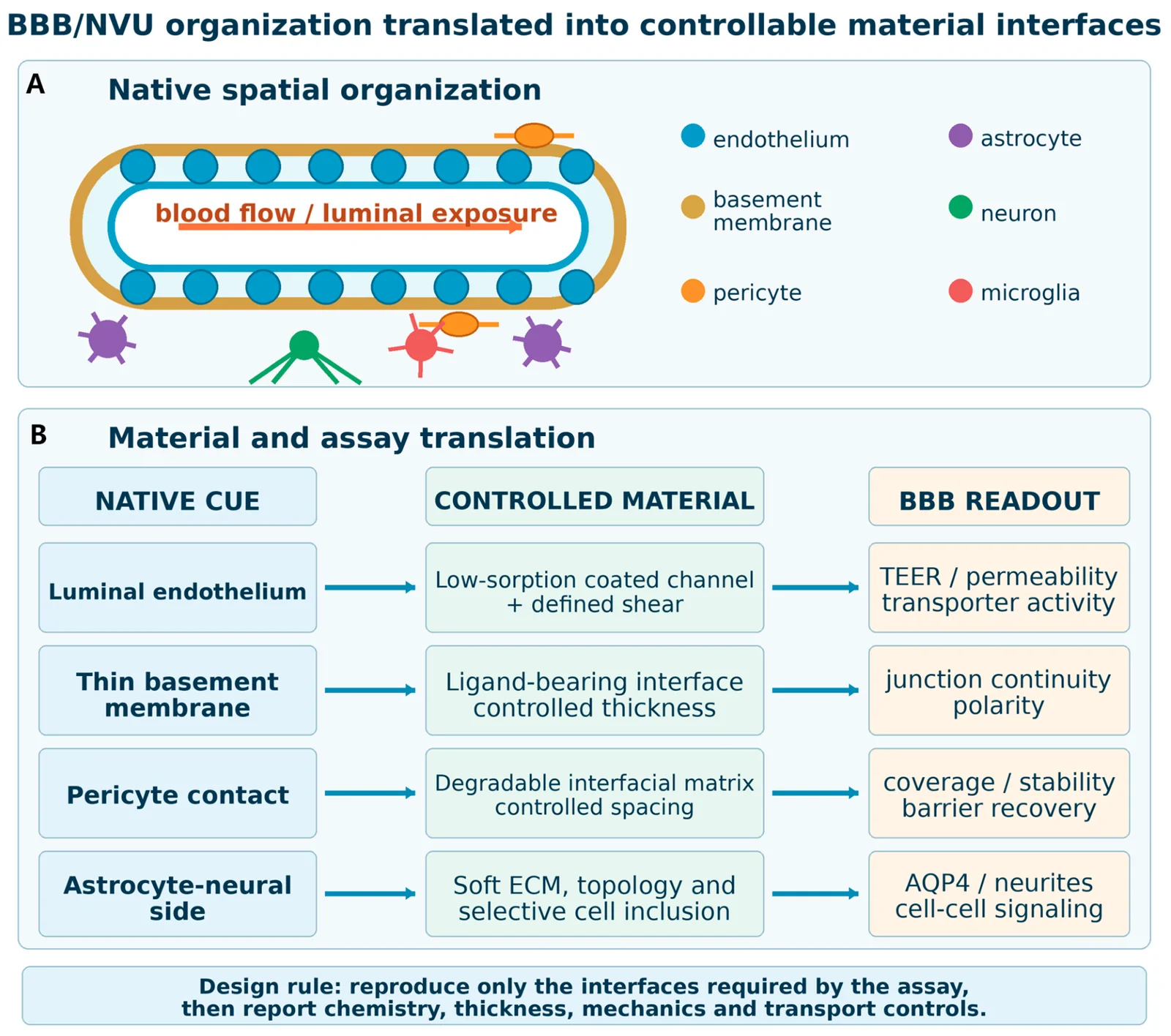
Schematic translating BBB/NVU spatial organization into material interfaces. (**A**) Native localization of endothelial cells, basement membrane, pericytes, astrocytes, neurons and microglia; (**B**) translation of selected native cues into controllable material variables and BBB readouts. Created by the authors based on the concepts summarized by Sweeney et al. [32]; no third-party graphical elements were reproduced.

BBB/NVU organization translated into controllable material interfaces shown in Figure 1. The endothelial–basement-membrane interface is the minimum material unit of a BBB model [49,50]. It requires a low-sorption luminal surface for brain microvascular endothelial cells and a thin, adhesive abluminal phase containing collagen-, laminin- or proteoglycan-related cues; development, junction organization, glycocalyx contributions and suppression of transcytosis all inform which functions that interface must reproduce [51,52,53,54,55]. Recent studies show that the support is not passive. Choi et al. used an engineered basement membrane to enhance barrier properties in a human iPSC-derived model [42]; Singh et al. embedded differentiated stem-cell-derived BBB components in collagen hydrogel [45]; and Augustine et al. used photocrosslinked GelMA to create a disease-oriented BBB construct [47]. Most recently, Sun et al. decellularized, defatted and lyophilized squid mantle into a porous scaffold film that supported endothelial cells and astrocytes and produced a reported TEER of approximately 230 Ω·cm^2^ with restricted 10 and 70 kDa dextran transport [43]. Together, these studies indicate that interface thickness, pore architecture, ligand identity, crosslinking and processing history must be treated as independent experimental variables rather than as background culture details [42,45,47].

Perivascular contact is the second material requirement, but recent research argues against simply adding more cell types [56,57,58]. Developmental and adult studies show that pericytes [59,60,61,62], mural-cell signaling [63] and astrocyte–endothelial communication [64,65,66] contribute differently to barrier induction and maintenance [59,67,68,69]. Campisi et al. used fibrin to support a self-organized endothelial–pericyte–astrocyte microvascular network [44]. Ahmad et al. instead used peptide-functionalized PEG to provide a chemically defined endothelial–astrocyte matrix [70]. Nakayama-Kitamura et al. then showed that collagen I microfibers promoted astrocyte survival through integrin-β1-associated interactions and increased capillary-network formation and maturation [46]. These results identify three distinct material levers—cell-driven fibrin remodeling, defined peptide presentation and collagen-fiber topography—but they cannot be ranked directly because cell source, geometry, mechanics and analytical endpoints differed. A review-level comparison should therefore ask which material variable was isolated and which variables remained confounded [46,71,72].

Microglia and neurons should be added only when the experimental question requires immune remodeling or neurovascular coupling [73,74,75,76]. Their inclusion demands degradable or neurite-permissive matrices and time-resolved controls that separate cell-driven remodeling from changes in tracer diffusion [77,78,79,80]. Studies of microglial mechanosensing [75,81], juxtavascular dynamics, purinergic control [82], endothelial-junction injury, reactive astrocytes and activity-dependent vascular patterning illustrate the distinct signals that a material may need to present or permit [81,83,84,85]. Maoz et al. demonstrated metabolic coupling in linked endothelial and neuronal compartments [9], whereas recent BBB hydrogel studies have generally prioritized endothelial–pericyte–astrocyte organization [45,46,47,70]. Thus, the principal design decision is not whether a model contains every NVU cell, but whether its luminal surface, interface thickness, perivascular matrix and cell-contact topology are appropriate for the claimed readout [12,16,41].

### 2.2. Gaps and Material-Focused Research Needs

The recent studies above also expose the principal literature gaps. First, fibrin, collagen, GelMA, PEG and dECM studies use different cells, geometries, flow conditions and endpoints, preventing head-to-head material ranking. Second, natural materials are still incompletely defined by source, lot, gelation kinetics and fiber architecture, while synthetic systems are often reported without ligand molarity, conversion, mesh size or extractables. Third, interface thickness and device stiffness are rarely matched to controls. Finally, barrier performance is variously reported as junctional fluorescence, TEER or a single tracer at different time points and areas [15,16,17,42,86]. The meta-analysis by Shamul et al. quantified this heterogeneity, while the engineered-basement-membrane and collagen-microfiber studies illustrate the stronger alternative of linking one defined structural intervention to a barrier or network outcome [12].

One clinically relevant target phenotype is early, low-level BBB leakage rather than catastrophic barrier failure. Nation et al. combined dynamic contrast-enhanced MRI with the cerebrospinal-fluid pericyte-injury marker soluble PDGFRβ and found hippocampal capillary damage in people with early cognitive dysfunction independently of amyloid-β (Aβ) and tau status [87]. Montagne et al. subsequently reported that baseline soluble PDGFRβ predicted future cognitive decline in APOE4 carriers and linked this phenotype to activation of the cyclophilin A–matrix metalloproteinase-9 pathway [88]. A prospective elderly cohort also associated higher regional BBB permeability with lower cognitive scores, although it did not identify excessive arterial pulsatility as the trigger [89]. Thus, “risk” here denotes an early vascular abnormality that is associated with, or predicts, later cognitive decline; it does not prove that BBB breakdown alone causes Alzheimer’s disease. Stroke and reperfusion studies likewise show that inflammatory signaling, MMP-associated junction redistribution and stage-dependent permeability can produce graded rather than all-or-none barrier failure [90,91,92]. The biological concern is that pericyte injury, junctional loss and basement-membrane degradation permit plasma proteins and inflammatory mediators to enter neural tissue, amplify glial inflammation, disturb ionic and metabolic homeostasis, and impair waste and Aβ clearance [40,93,94]. Biomaterial BBB models intended for Alzheimer’s research should therefore resolve mild permeability changes, pericyte injury and matrix degradation, rather than validate only gross leakage.

Future studies should therefore use factorial designs that vary chemistry, stiffness, degradability and cell composition independently; report full preparation histories; and combine TEER or a molecular-size permeability series with junctional and transporter markers. Acellular controls are needed whenever a hydrogel trigger, degradation event or secondary network can change diffusion independently of endothelial behavior. These practices would convert Section 2 from descriptive anatomy into testable biomaterials criteria and enable meaningful comparison of natural, synthetic and hybrid BBB matrices [12,15,16,17,41].

## 3. Hydrogel Biomaterials for BBB Models: Chemistry, Preparation, and Functionalization

A hydrogel is not a single material but a water-swollen, three-dimensional network of hydrophilic polymer chains that is prevented from dissolving by covalent crosslinks or reversible physical interactions. Its chemical formulation comprises (i) one or more polymer backbones, (ii) crosslinkable or associating groups, (iii) a crosslinker, initiator, enzyme or counter-ion when required, (iv) water or culture medium and, for cell-instructive systems, (v) adhesive peptides, degradable sequences, extracellular-matrix proteins or particles [95,96,97]. Polymer identity, molecular weight, concentration, functionalization and crosslink density jointly determine stiffness, mesh size, swelling, degradation, ligand availability and solute diffusion; these variables can change an apparent BBB permeability result even when endothelial junctions do not change [98,99,100].

Chemically, natural protein hydrogels include collagen I, fibrin and gelatin or gelatin methacryloyl (GelMA); natural polysaccharide systems include hyaluronic acid or hyaluronic-acid methacrylate (HAMA), alginate and chitosan; dECM hydrogels contain a tissue-derived mixture of proteins and glycosaminoglycans; and synthetic systems include PEG, PVA, polyacrylamide and PNIPAM [47,86,101]. Natural matrices usually supply cell-binding and protease-remodelable motifs, but their source, lot, fibril structure, contraction and weak mechanics reduce reproducibility [45,46]. Synthetic matrices provide defined and independently tunable chemistry, but PEG, PVA and polyacrylamide are intrinsically nonadhesive and require deliberate addition of ligands and degradable crosslinks [70,102,103]. dECM offers complex tissue cues but is compositionally uncertain and process dependent. Hybrid and interpenetrating networks can balance bioactivity with shape retention, although mixed crosslinking reactions make causal attribution, sterilization and quality control more difficult [104,105,106].

### 3.1. Hydrogel Chemistry, Preparation, and Peptide Functionalization

**Figure 2:**
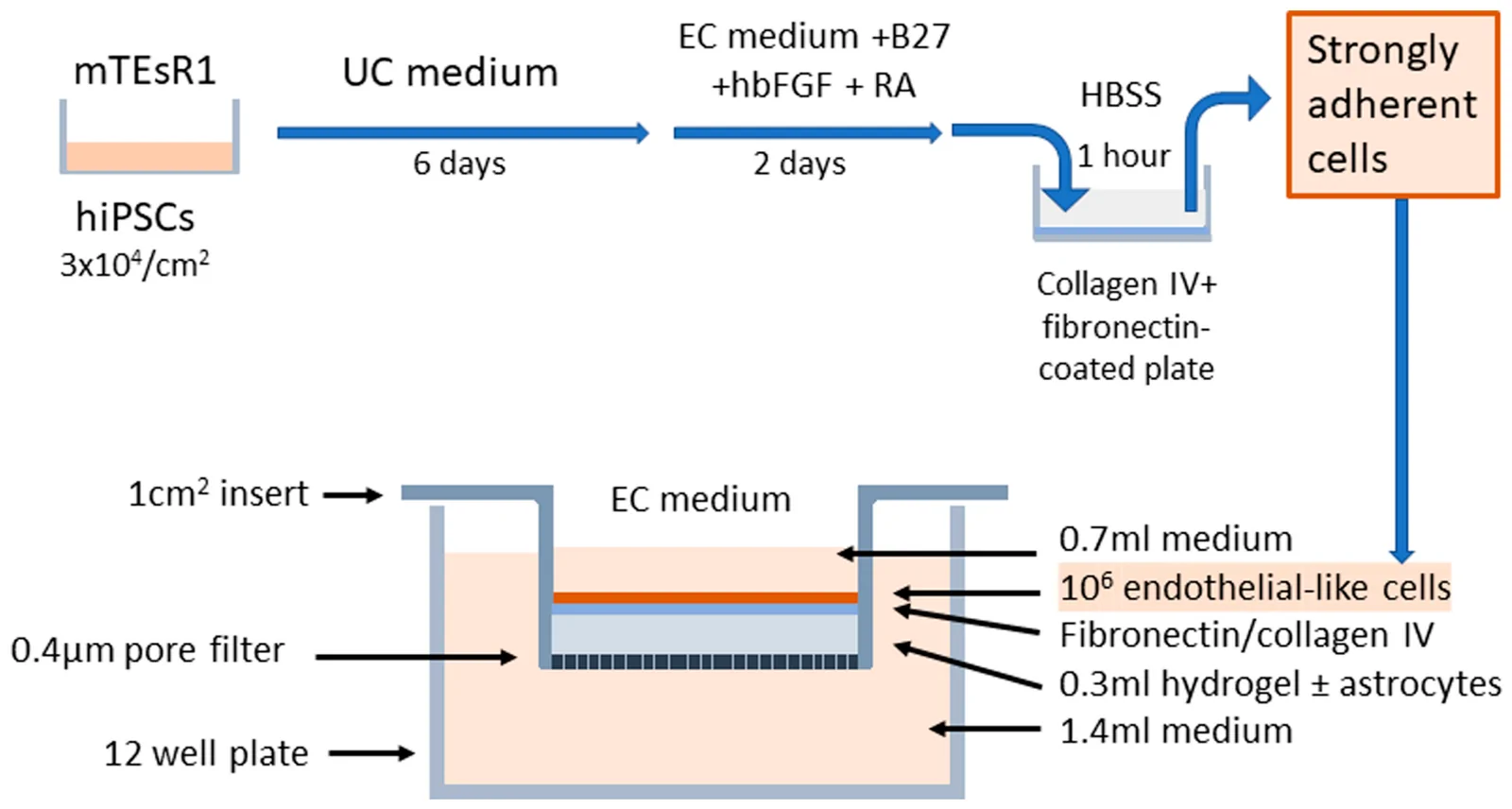
Example of collagen I hydrogel preparation for a human BBB model. The original schematic shows hiPSC differentiation, the 0.3 mL collagen I hydrogel with optional astrocytes, the collagen IV/fibronectin surface layer and endothelial seeding in a Transwell plate. Reproduced from Singh et al. [45] under the Creative Commons Attribution license.

Preparation starts by dissolving or dispersing polymer precursors, cells and optional ligands under cytocompatible conditions, followed by network formation. Collagen I is diluted and neutralized on ice and then warmed to drive fibrillogenesis; fibrin forms when thrombin cleaves fibrinogen; alginate crosslinks ionically with Ca^2+^; and chitosan/β-glycerophosphate gels by temperature-dependent physical association (Figure 2). GelMA and HAMA are first produced by methacrylation, purified and lyophilized, then redissolved with a photoinitiator and crosslinked by controlled light exposure. Multi-arm PEG can be crosslinked by acrylate photopolymerization, thiol–ene coupling or Michael-type addition, whereas dECM hydrogels require tissue decellularization, washing, lyophilization, milling, enzymatic digestion and neutralization before thermal gelation [47,104,105,106]. Polymer concentration, degree of substitution, precursor stoichiometry, initiator dose, wavelength, exposure, pH and temperature are therefore part of the material definition, not incidental protocol details.

**Figure 3:**
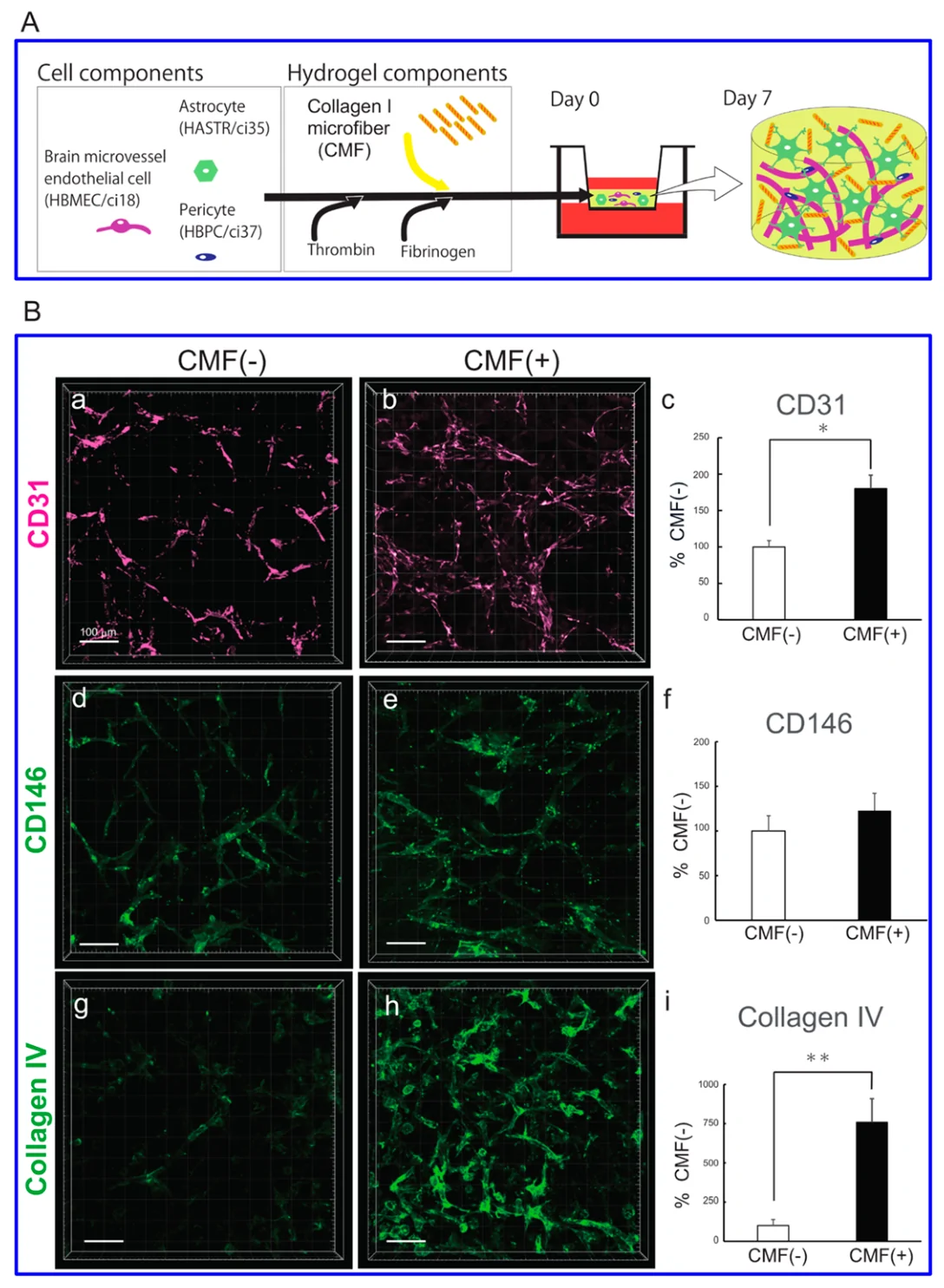
Comparison of fibrin hydrogels with and without collagen I microfibers, including the preparation scheme, vascular-marker images and quantified capillary-network outcomes. (**A**) Schematic of the BBB–NET components. (**B**) Representative z–stack images of the endothelial cell markers CD31 (**a**,**b**) and CD146 (**d**,**e**) and the basement membrane protein collagen IV (**g**,**h**) in hydrogels with (**b**,**e**,**h**) and without (**a**,**d**,**g**) CMFs. Scale bar: 100 μm. Comparison of the capillary volumes positive for CD31 (**c**), CD146 (**f**), and collagen IV (**i**) among hydrogels with and without CMFs. Student’s *t* test, *: *p* < 0.05, **: *p* < 0.01. Reproduced from Nakayama-Kitamura et al. [46] under CC BY 4.0.

Bioactive peptides are incorporated to restore specific cell–matrix interactions or degradability. RGD supports integrin-mediated adhesion, IKVAV and YIGSR reproduce laminin-derived signals, and matrix metalloproteinase (MMP)-cleavable sequences permit cell-directed remodeling. Ahmad et al. co-cultured endothelial cells and astrocytes in a peptide-functionalized PEG hydrogel, demonstrating that a chemically defined synthetic matrix can be made permissive to BBB-relevant adhesion and communication [70]. PEG is therefore one design family rather than the defining example: collagen and fibrin provide native adhesion sites without peptide grafting [44,45], GelMA retains gelatin-derived motifs while allowing photocrosslinking [47,105], and collagen microfibers add vascular-scale topography and integrin engagement (Figure 3) [46].

Singh et al. provide a preparation-to-function example for collagen I. A 5 mg/mL rat-tail collagen I solution was made isotonic, neutralized with NaOH, mixed with astrocytes and allowed to gel for approximately 10 min at 37 °C in a Transwell; the surface was subsequently coated with collagen IV/fibronectin before seeding hiPSC-derived endothelial-like cells [45]. The resulting model reached TEER values above 700 Ω·cm^2^, restricted paracellular tracers and displayed functional transferrin-receptor and ABCB1 transport. Its useful transport window was nevertheless only about 24–72 h because TEER declined over time, and the hydrogel cultures had lower resistance and slightly higher paracellular permeability than matched endothelial monolayers on filters [45]. This example illustrates both the bioactivity and accessibility of collagen and the need to report concentration, geometry, time window and contraction rather than label a formulation simply as “collagen hydrogel.”

Nakayama-Kitamura et al. compared fibrin alone with a fibrin–collagen I microfiber composite, rather than treating all natural hydrogels as equivalent. They mixed 0.7 mg collagen I microfibers and 0.4 mg fibrinogen with endothelial cells, astrocytes and pericytes, added 0.3 U thrombin and gelled the construct for 45 min before 7-day culture [46]. Relative to the microfiber-free gel, collagen I microfibers increased CD31-positive capillary volume to 181.9%, collagen IV-positive capillary volume to 758.8% and astrocyte number to 174.1%; integrin-β1 blocking reduced astrocyte survival. The advantages were added contact guidance, astrocyte support and capillary maturation; the limitations were a multicomponent formulation, dependence on approximately 20 μm fiber geometry and the need for microfiber quality control [46].

A 2025 study extended this comparative strategy to a soft synthetic–natural hybrid. DePalma et al. first found that thiolated hyaluronic acid (HA) plus GelMA was too soft to retain a cylindrical lumen; adding collagen I improved endothelial attachment, but the vessels constricted and collapsed after 4–6 days, while adding laminin–entactin supported attachment for at least 6 days but did not produce stellate astrocytes [107]. Their final formulation contained 0.05% (*w*/*v*) thiol-HA, 1.5% GelMA, 15% ionically crosslinked VitroGel-IKVAV and 0.1% Irgacure 2959. It was incubated for 10 min at 37 °C, exposed to 365 nm light for 2 s and then completed ionic crosslinking in medium. The approximately 300 Pa matrix supported a stable lumen, stellate astrocytes, endothelial tight junctions and low small-molecule permeability for at least 10 days [107]. This sequence of unsuccessful and successful formulations is especially informative because it identifies the trade-off among lumen stability, endothelial adhesion and astrocyte phenotype instead of reporting only the final material. Representative hydrogels, including their incorporated ligands/peptides, preparation, BBB/NVU applications and limitations, are summarized in Table 2.

### 3.2. Stimuli-Responsive Hydrogels: Mechanisms and BBB Relevance

The hydrogels discussed in Section 3.1 are usually designed to retain approximately fixed properties after curing. A stimuli-responsive hydrogel is different: its network contains an ionizable group, a cleavable or reversible bond, a phase-transitioning polymer, or an embedded functional particle that is deliberately converted by a defined signal. The common mechanism is therefore a four-step materials sequence: (i) a thermal, biochemical, optical, acoustic, electrical or magnetic trigger is recognized; (ii) polymer solvation, bond state, particle polarization or supramolecular association changes; (iii) the network changes its crosslink density, mesh size, swelling, modulus or degradation rate; and (iv) that change produces a measurable transport, release or cell–matrix response [48,98]. Calling a gel “responsive” consequently requires a reversible or programmed post-trigger material change, not merely the use of heat or light during its initial fabrication.

**Figure 4:**
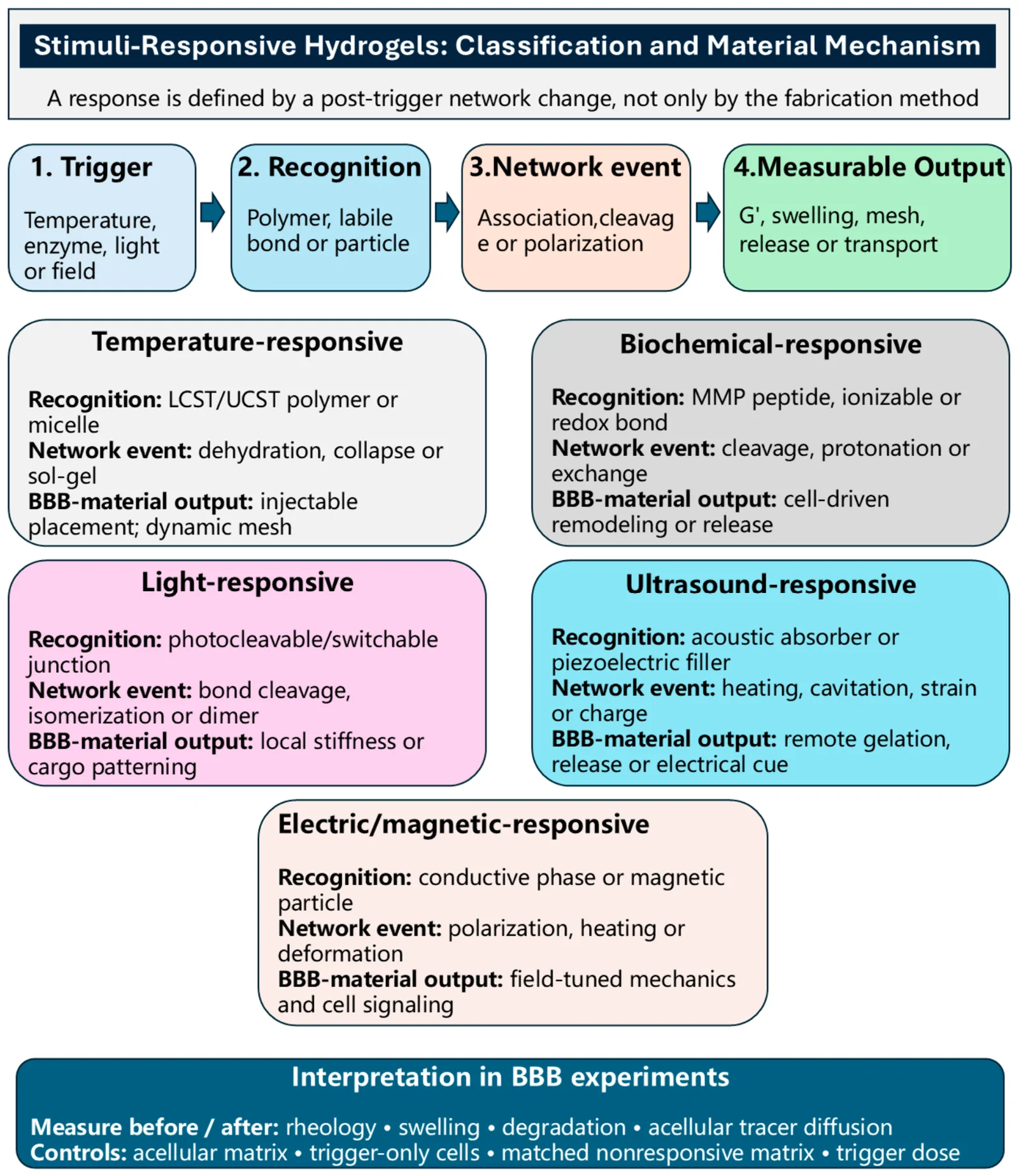
Materials-science classification of stimuli-responsive hydrogels. Every category follows the same causal chain—trigger, recognition element, network event and measurable output—but only BBB experiments with material and trigger controls can distinguish a genuine barrier response from altered diffusion through the gel [48,86,98,99,108,109,110,111]. Original schematic created for this review; no third-party graphical elements were reproduced.

This signal-to-network mechanism is relevant to BBB biomaterials at three stages. During fabrication, a low-viscosity precursor can gel around endothelial, pericyte or astrocyte compartments without mechanically disruptive molding. During culture, dynamic stiffness, ligand exposure or enzymatic remodeling can model changing basement-membrane conditions [41,48]. During permeability experiments, controlled mesh opening or cargo release can impose a timed perturbation. The same responsiveness also creates a confounder: swelling, erosion or trigger-induced heating can change tracer diffusion even when endothelial junctions are unchanged. Each BBB experiment therefore requires an acellular material control, a trigger-only cell control, and pre/post-trigger measurements of rheology, swelling and molecular permeability. Direct in vitro BBB validation remains sparse; most recent reports concern brain implants, organoids, regenerative delivery or other vascularized tissues, so they establish material mechanisms but not automatically barrier fidelity [48,98]. Figure 4 organizes these findings by recognition chemistry and network output rather than by medical indication.

Thermoresponsive hydrogels undergo a temperature-dependent sol–gel or volume-phase transition. In a lower-critical-solution-temperature (LCST) system, polymer–water hydrogen bonding dominates below the transition; heating increases hydrophobic association and chain dehydration, producing collapse, micellization or gelation. Upper-critical-solution-temperature systems show the opposite trend [48,86]. PNIPAM, whose homopolymer transition is near 32 °C, is commonly prepared by free-radical polymerization with a multifunctional crosslinker, by controlled radical polymerization followed by grafting, or as a copolymer/composite that shifts the transition toward physiological temperature [108]. Poloxamers and chitosan/β-glycerophosphate are mixed cold and physically associate on warming, whereas covalently crosslinked PNIPAM networks retain shape more strongly but may show syneresis and limited degradability. Thus, preparation method controls not only gelation temperature but also hysteresis, leaching and long-term dimensional stability [86].

Two recent temperature-responsive studies illustrate why these synthesis variables must be reported. Belyaeva et al. synthesized PNIPAM by a controlled radical route and grafted it to cellulose nanocrystals; the resulting CNC–g–PNIPAM dispersion gelled near body temperature, supported primary brain microvascular endothelial cells and astrocytes, and released paclitaxel, but it was evaluated as a brain-compatible implant rather than a perfused BBB model [86]. Promdontree et al. instead combined 5% N-isopropylacrylamide, chitosan, 0.1–3% cellulose nanocrystals and Irgacure 2959, removed oxygen under nitrogen and photopolymerized the composite for 30 min. The formulations showed volume-phase-transition temperatures of 34–38 °C and an increase in storage modulus above 30 °C [108]. This study provides a reproducible preparation benchmark, although it did not use BBB cells. For BBB use, temperature-responsive candidates should therefore be compared at matched transition temperature, heating/cooling rate, gelation time, storage modulus, swelling, syneresis and permeability rather than being grouped simply as “thermogels.”

Biochemically responsive networks translate local chemistry into remodeling. Matrix metalloproteinase (MMP)-sensitive peptides are cleaved to enlarge the mesh or release a tethered factor; protonatable carboxyl or amine groups change ionization and swelling with pH; and disulfide or thioketal units respond to reducing or oxidative conditions. Liu et al. recently self-assembled peptide-amphiphile nanofibers containing the MMP-2-cleavable sequence PLGLAG and the VEGF-mimetic QK peptide. MMP-2 increased QK release, and the material promoted endothelial migration and tube formation; in a rat cerebral ischemia/reperfusion model it increased VE-cadherin and ZO-1 and reduced BBB leakage [109]. This provides stronger BBB-related evidence than a generic delivery study does, but it remains an in vivo repair system rather than an in vitro barrier platform. The material advantage is cell- or disease-coupled feedback; the corresponding limitation is that enzyme concentration, pH or redox state simultaneously changes gel diffusion. Trigger-free and cell-free release/permeability curves are therefore essential.

Externally actuated gels offer a different advantage because the operator selects when and where the stimulus is applied. A light-responsive hydrogel must change after gel formation; a GelMA network that is merely photocured is photofabricated but is not necessarily light-responsive. Here, “high spatiotemporal control” has two specific materials-science meanings. Spatial control means that a focused or projected beam, or a photomask, confines bond cleavage, photoisomerization or additional crosslinking mainly to the illuminated region; neighboring regions can retain their original mesh size and modulus. Temporal control means that switching the light on and off defines the onset and exposure window, while pulse number and total dose can tune the extent of conversion without adding a soluble reagent to the entire culture. Fan et al. functionalized hyaluronic acid with Brooker’s merocyanine guests and crosslinked it supramolecularly with cucurbituril (CB8). Masked UV exposure drove [2+2] photodimerization, locally converted reversible junctions to covalent ones and spatially tuned viscoelasticity and lymphatic tube formation [110]. Afting et al. formed DNA hydrogel microbeads from Y-motif strands and photocleavable linkers in water-in-oil droplets; 405 nm irradiation disassembled selected beads and generated localized Wnt-surrogate gradients in retinal organoids [111]. Thus, “high” is a relative rather than unlimited claim: spatial resolution is constrained by optical focusing, light absorption and scattering, gel thickness and diffusion of radicals or photoproducts, whereas temporal resolution is constrained by photoreaction kinetics, network relaxation and post-release cargo diffusion. Neither study is a BBB validation, and wavelength, intensity, dose, oxygen inhibition, photoproducts and post-exposure mechanics would require independent assessment in BBB cells [111].

Ultrasound-responsive composites convert acoustic loading into heat, cavitation, deformation or piezoelectric charge. Wang et al. dispersed KNN piezoelectric nanocrystals and reduced graphene oxide in a gelatin/PVA matrix; ultrasound enhanced electrokinetic and angiogenic responses, but the model addressed neurovascular regeneration in diabetic wounds rather than BBB transport [99]. Conductive or magnetic fillers can likewise produce field-dependent charge, heating or deformation. For a BBB-oriented material study, acoustic pressure or field strength, duty cycle, temperature rise, filler release, cytotoxicity and barrier permeability must be measured together. The studies in Table 3 therefore separate demonstrated material response from BBB-specific findings instead of treating all responsive hydrogels as clinically interchangeable.

Across these studies, the strongest materials concern synthesis–structure–property relationships, whereas direct in vitro BBB evidence remains lacking. A useful next experiment would compare one responsive and one nonresponsive matrix at matched baseline modulus and ligand density, apply the same trigger, and quantify network mechanics, acellular tracer diffusion, TEER, size-dependent permeability, junction continuity and transporter function over the same time course. This control hierarchy connects dynamic-material design to interpretable BBB biology and leads naturally to the composite and interpenetrating networks discussed next, where multiple phases are combined to decouple mechanics, bioactivity and stimulus recognition.

### 3.3. Composite and Interpenetrating-Network Hydrogels

A composite hydrogel is obtained by combining a primary water-swollen network with at least one second material phase whose function can still be identified. Four preparation routes are common [103]. In one-pot blending, a soluble polymer or dispersed fiber, microgel or particle is mixed with the primary precursor before a single gelation step. In orthogonal co-crosslinking, two compatible reactions proceed in the same sample, such as thrombin-mediated fibrin formation together with photopolymerization of PEGDA or GelMA [47,95]. In sequential, network-by-network fabrication, the first gel is formed, a second precursor is allowed to diffuse through it, and the second network is then crosslinked. Preformed fibers or microgels can also be embedded mechanically in an already defined precursor. Interpenetrating polymer networks (IPNs) contain two continuous crosslinked networks, whereas semi-IPNs contain one crosslinked network and one entangled linear polymer. Mixing order, precursor viscosity, crosslinking-rate matching and chemical compatibility determine whether the final material is homogeneous, phase-separated or anisotropic [101,102,103].

The property gained depends on the added phase and should not be described simply as a generic “composite improvement.” PEGDA or another covalent synthetic network usually increases dimensional stability, resistance to cell-mediated contraction and the range over which modulus can be tuned. Collagen, fibrin or gelatin supplies integrin-binding and protease-remodelable sites that bioinert PEG lacks. Hyaluronic acid increases water retention and CNS-ECM biochemical relevance but can also reduce permeability when densely packed [46]. Fibers introduce anisotropy and contact guidance, whereas microgels can create interconnected macropores; conductive, piezoelectric or magnetic particles add field responsiveness but may increase heterogeneity and change diffusion [101,102,103,107].

Diaz-Lasprilla et al. provide a direct example of how such a material is obtained. They used a network-by-network method for collagen I/fibrin/HA/PEGDA mIPNs: fibrinogen was mixed with the collagen–HA precursor, thrombin generated the fibrin network, collagen fibrillogenesis proceeded for 30 min at 37 °C, and PEGDA chains were subsequently polymerized by UV exposure [102]. Increasing fibrin produced a denser fibrous network and increased the complex modulus; PEGDA molecular weight and concentration were adjusted to improve physical stability and fine-tune the modulus, while HA retained a brain-ECM-related biochemical variable. Formulations containing 3.0 mg/mL collagen I, up to 6.0 mg/mL fibrin, 2.0 mg/mL HA and 3.4–10.0 kDa PEGDA approached the approximately 7.35 kPa complex modulus of the selected diseased-cortex reference and supported at least 95% astrocyte viability after 24 h [102]. This study demonstrates compositional and mechanical tunability, but it is a CNS scaffold rather than a validated BBB permeability model.

Two BBB-oriented studies show how the second phase changes a measurable outcome. Nakayama-Kitamura et al. mixed 0.7 mg collagen I microfibers with 0.4 mg fibrinogen, combined this precursor with endothelial cells, astrocytes and pericytes plus 0.3 U thrombin, and gelled it for 45 min [46]. Relative to fibrin without microfibers, the added collagen phase supplied topographical and adhesion cues and increased CD31-positive capillary volume to 181.9%, collagen IV-positive capillary volume to 758.8% and astrocyte number to 174.1% [46]. DePalma et al. used a different hybrid route: thiolated HA and GelMA were photocrosslinked, while IKVAV-functionalized VitroGel completed ionic crosslinking in culture medium [107]. Relative to the softer HA/GelMA trial formulations, incorporation of the ionically crosslinked, IKVAV-bearing phase was associated with improved lumen shape retention and cell–matrix support; the final approximately 300 Pa matrix maintained a stable lumen, stellate astrocytes, endothelial junctions and low small-molecule permeability for at least 10 days [107].

These examples also define the necessary controls. A stiffer or more stable composite is not automatically a better BBB matrix: added crosslinks can reduce nutrient or tracer diffusion, fibers can create local anisotropy, and particles or mixed curing reactions can introduce leachables and spatially nonuniform properties [46,101]. Studies should therefore compare the primary gel with each added phase and with the final composite at matched cell density, report mixing order and curing conditions, and measure rheology, swelling, microstructure and acellular permeability before attributing an endothelial response to the composite formulation [102,107].

### 3.4. Multi-Material Integration on a Chip

A BBB device often requires a rigid, optically accessible perfusion body, a thin vascular interface and a softer parenchymal hydrogel. Multi-material fabrication addresses this mismatch through sequential casting, surface bonding, sacrificial templating, embedded printing or multi-nozzle bioprinting. A recent platform used materials of graded stiffness to create perfusable microvasculatures, illustrating how rigid channel supports and cell-compatible matrices can be integrated rather than forced into one formulation [101]. The interface between materials remains the critical failure point: delamination, uncured oligomers, differential swelling and nonspecific sorption can change flow or transport [17,100]. Consequently, bonding chemistry, extractable testing, swelling under culture conditions and interfacial leakage should be reported together with biological results [101].

These hydrogel routes should be selected by experimental context rather than by biological complexity alone. Neutralized collagen and thrombin-crosslinked fibrin are inexpensive, cytocompatible and easy to cast in parallel, making them attractive for exploratory cell–matrix studies; their lot variability, contraction and degradation make long-term standardization difficult [46]. PEG and peptide-defined networks offer higher chemical reproducibility and are more compatible with factorial or multiwell screening, but synthesis, purification and ligand stoichiometry increase development effort, and unmodified networks are bioinert [45,96]. GelMA and HAMA occupy an intermediate position: photocuring supports patterning and batch fabrication, yet light dose, initiator and degree of substitution must be controlled [47]. Stimuli-responsive and composite/IPN systems are most useful when dynamic remodeling, release or mechanical reinforcement is the question; because they add triggers, crosslinking steps and transport confounders, they are currently less suitable for routine high-throughput or regulated screening [70,99]. DLP improves geometric repeatability and parallel feature generation, whereas extrusion/coaxial printing enables higher cell density and multi-material placement but has lower resolution and greater shear-related variability [46,48,105]. Thus, reproducibility, scale, cost and long-term stability generally favor simpler defined systems, while mechanistic fidelity and spatial control may justify more complex fabrication.

## 4. Fabrication Strategies for Engineered In Vitro BBB Models

In vitro BBB models serve as indispensable tools for diverse scientific applications, including mechanistic research, identification of therapeutic candidates in neuroscience, and high-throughput drug screening [112]. Substantial advances have been achieved to refine these in vitro models in recent years, enabling the formation of compact 2D monolayers, tubular 2D architectures, and even complex microvascular networks in certain platforms [113]. It must be noted that the BBB constitutes a dynamic interface continuously exposed to fluid shear stress derived from blood flow [114]. The latest generation of human in vitro BBB recapitulation platforms incorporates fluid perfusion to mimic physiological hemodynamic conditions, thereby reproducing native BBB characteristics with enhanced fidelity. Accordingly, such in vitro systems are categorized into static and dynamic models based on their capacity to deliver fluid flow and impose shear stress. Static culture systems retain cells submerged in culture medium without perfusion or continuous flow, allowing only mild fluctuations in the extracellular microenvironment [115]. In contrast, dynamic models utilize pumping modules to generate steady perfusion with defined pressure and flow rates, recapitulating more pronounced physiological microenvironmental variations. Fluid flow and shear stress are critical for sustaining authentic BBB phenotypes within in vitro constructs; therefore, dynamic culture platforms mimic the human BBB more accurately [116].

An ideal in vitro BBB model should faithfully recapitulate the following core in vivo features of the native barrier [56,117,118]: (1) 3D vessel-like tubular structures formed by endothelial cells and ensheathed by pericytes and astrocytes. (2) Tri-dimensional co-culture of principal neurovascular cell populations, including endothelial cells, pericytes, astrocytes, microglia and neurons. (3) Robust cell–cell crosstalk between all constituent cell types. (4) Physiological fluid shear stress applied to endothelial monolayers. (5) Thin basement membrane (BM) with selective macromolecular permeability.

This section systematically introduces engineering approaches for constructing in vitro BBB models and summarizes the respective merits and limitations of each strategy. Current engineered BBB platforms fall into four major categories: 3D cellular spheroid models, Transwell-based systems, microfluidic BBB chips, and 3D-bioprinted BBB constructs [112,113,117].

### 4.1. Three-Dimensional Spheroid Models

Human BBB organoids are self-assembled 3D architectures generated via the migration, proliferation and spontaneous organization of microvascular cells, wherein NVU cell populations reconstruct native three-dimensional cytoarchitecture [119,120]. Significant progress has been made in generating BBB-mimetic organoids that recapitulate hallmark barrier phenotypes, including abundant expression of intercellular junction proteins, transporters and carrier proteins, laying a solid foundation for future applications in BBB drug transport and toxicological assessments [121,122].

Cho et al. seeded brain microvascular endothelial cells, brain microvascular pericytes and astrocytes onto low-adhesion extracellular matrix substrates to generate compact solid 3D spheroids. Within these constructs, an astrocyte core linked by intercellular junction proteins is surrounded by an outer layer of endothelial cells and pericytes [121,122]. Separately, Nzou et al. established 3D spheroids incorporating human brain microvascular endothelial cells, human brain pericytes, human astrocytes and human neurons [123]. The defining advantage of spheroid models lies in the extensive, direct cell–cell contact established among all cell subsets within the aggregate, which is critical for preserving BBB integrity and physiological function.

Despite major breakthroughs in spheroid BBB engineering, these systems possess notable limitations in recapitulating native BBB structure and function [124].

First, real-time detection and tracking of the spheroid core are technically challenging. The solid core structure creates a major bottleneck for permeability assays, as molecular transport into the central region cannot be readily quantified or visualized [122,123,124].

Second, physiological blood flow and shear stress cannot be recapitulated. Since existing BBB organoids are maintained under static culture, endothelial barrier interfaces are not subjected to physiological perfusion or shear stimuli, hindering the formation of a tight endothelial barrier. Although genetic and phenotypic modification of BBB cell populations can modulate endothelial barrier properties, such strategies are restricted by high costs, complicated workflows and prolonged culture cycles [114,116].

Furthermore, poor batch-to-batch reproducibility represents the primary obstacle limiting widespread adoption of spheroid platforms [12,124].

### 4.2. Transwell-Based Platforms

The Transwell format consists of apical and basolateral compartments separated by a porous polymer membrane, commonly polycarbonate (PC), polyethylene terephthalate (PET) or polytetrafluoroethylene (PTFE) [125]. It should therefore be understood as a biomaterial system rather than a neutral cell holder. The membrane polymer, thickness, pore diameter and porosity determine the available adhesion area, the distance between endothelial and perivascular cells, and the resistance added to molecular transport [126,127]. Representative Transwell and membrane-supported BBB studies, with their biomaterial variables and reported consequences, are summarized in Table 4. Collagen IV, fibronectin, laminin or poly-L-lysine coatings change integrin-mediated adhesion, while a collagen, Matrigel, GelMA or peptide-functionalized PEG layer adds a three-dimensional matrix whose stiffness, mesh size and degradation alter cell phenotype and tracer diffusion [45,47,128].

Stone et al. directly demonstrated that the insert is an experimental material variable. Their four-primary-human-cell model used 12 mm collagen-coated inserts; the basolateral surface was further coated with 2 μg/cm^2^ poly-L-lysine before astrocyte attachment [127]. Increasing pore size from 0.4 to 3.0 μm increased TEER and improved contact among endothelial cells, astrocytes and pericytes. Thus, the apparent barrier improvement did not arise only from adding cell types: pore geometry and surface chemistry changed cell proximity and adhesion [127]. The larger pores nevertheless increase the possibility of cell crossing and make the membrane less comparable to a continuous native basement membrane.

Kim et al. created a simplified 3D Transwell co-culture by mixing immortalized human brain endothelial cells and astrocytes with Matrigel, allowing assembly within approximately 30 min [126]. Matrigel provides rapid thermal gelation and a mixture of basement-membrane proteins, but its tumor-derived, compositionally undefined and lot-dependent character limits attribution of the barrier response to a specific ligand or modulus. In contrast, Singh et al. placed approximately 0.3 mL of 5 mg/mL collagen I hydrogel, optionally containing primary human astrocytes, in a 0.4 μm insert; the gel surface was coated with collagen IV/fibronectin before seeding hiPSC-derived endothelial-like cells [45]. The resulting model reached TEER values above 700 Ω·cm^2^ and supported low paracellular permeability and transporter assays, although TEER declined over 10 days and the most reliable assay window was 24–72 h [45]. The collagen layer added a cell-remodelable 3D compartment, but its approximately 3 mm diffusion path produced a lag before tracer appearance, illustrating why hydrogel thickness must be included in permeability calculations.

Harding et al. retained a standard Transwell insert but mounted it in a custom millifluidic device, enabling endothelial–astrocyte–pericyte co-culture under physiological shear together with permeability, immunostaining and protein analyses [125]. This hybrid approach separates two design variables: the insert and coating remain the cell-contacting biomaterials, whereas the external housing supplies the mechanical stimulus. Choi et al. pursued the complementary material strategy of replacing a conventional thick insert interface with an ultrathin ECM-hydrogel engineered basement membrane supported by a sparse electrospun nanofiber scaffold [42]. The approximately 500 kPa interface supported iPSC-derived brain endothelial cells with strong physical barrier and efflux-pump functions, showing that membrane thickness, stiffness and ECM composition can be engineered together rather than treated as fixed properties [42].

Together, these studies establish the connection between Transwell models and biomaterials: the polymer insert provides geometry and mechanical support; the adsorbed protein layer provides adhesion signals; an added hydrogel provides three-dimensional mechanics and diffusion resistance; and a perfusion adapter provides shear. These contributions must be separated experimentally [12,42]. At minimum, studies should report membrane polymer, pore size, porosity and thickness; coating identity and surface mass; hydrogel composition, concentration, modulus and gelation method; and acellular permeability of the complete insert–coating–gel assembly [125]. Empty-insert, coating-only and gel-only controls are needed before a change in TEER or tracer flux can be assigned to endothelial biology [45,126]. The main advantage of the platform is controlled comparison with standard TEER and sampling workflows; its persistent limitation is the thick, rigid, planar separator, which prevents direct recapitulation of a thin compliant basement membrane or cylindrical vessel [127,128].

### 4.3. Microfluidic BBB Chips

Microfluidic BBB chips couple a perfused endothelial compartment to one or more perivascular compartments through a molded wall, porous membrane, hydrogel interface, cylindrical lumen or self-assembled vessel [11,15]. Regardless of category, the structure contains five functional modules: inlet/outlet reservoirs, a vascular flow path, a neural or stromal compartment, a selective interface, and access for imaging, sampling, electrodes or tracer analysis [16]. The interface may be a PC/PET membrane, a micropillar-confined gel wall, the surface of a templated ECM lumen or a cell-assembled capillary network [41,129]. Architecture therefore determines cell–cell distance, diffusion path, wall shear, sampling access and which biomaterial is actually in contact with each cell population [130,131,132].

A rational design proceeds from the measurement rather than from the chip material. TEER and paired luminal/abluminal sampling favor a membrane-separated device; real-time imaging of direct contact favors adjacent gel lanes; controlled circumferential shear favors a templated cylindrical lumen; and angiogenesis or capillary-network questions favor cell-driven self-assembly [15,16]. Channel dimensions and flow rate are then selected to reach the target wall shear without excessive pressure or gel deformation [16,41]. The device body, interface, ECM phase and surface coating are chosen separately, after which the seeding order is specified: perivascular cells are embedded or attached first when necessary, endothelial cells are allowed to form a confluent luminal layer, and perfusion is increased gradually. Empty-device leakage, acellular material permeability and coating stability under flow should be established before interpreting cellular barrier data [41,129,130].

In this context, “biomaterials” includes four functional classes rather than hydrogels alone. First, load-bearing device materials include PDMS, glass, PMMA and low-sorption cyclic olefin polymers (COC/COP) [15,16,17]; Ahn et al., for example, used plasma-bonded PDMS layers and a glass support [11]. Second, separation or basement-membrane-mimicking interfaces include PC, PET and PTFE membranes as well as electrospun PCL, PLGA or related nanofiber supports [9,42,133,134,135,136,137,138]. Ahn et al. used a 7 μm PC membrane, whereas Choi et al. supported an ultrathin ECM-hydrogel basement membrane with a sparse electrospun nanofiber network [11,42]. Third, cell-contacting coatings and 3D matrices include collagen I/IV, laminin, fibronectin, fibrin, hyaluronic acid, Matrigel, GelMA and peptide-functionalized PEG [44,45,47]. Representative examples are Bang et al.’s fibrin vascular-network matrix, Campisi et al.’s fibrin tri-culture, Singh et al.’s collagen I hydrogel and Wevers et al.’s collagen I gel with Matrigel coating [45,131,132]. Fourth, processing or sacrificial biomaterials include alginate and GelMA for printed lumens and gelatin or Pluronic as removable templates [24,130,139,140,141,142,143,144,145]. Table 5 compares these classes by architecture and experimental role.

These classes interact with cells through different mechanisms. The rigid device body defines channel geometry, optical access, oxygen transfer and drug sorption but should not be assumed to reproduce ECM biology [15,16,17]. Protein coatings and natural gels provide integrin-binding motifs, permit endothelial spreading and allow pericytes or astrocytes to remodel the matrix; brain endothelial cells also reorganize junctions under flow [41,44]. Synthetic PEG, PVA or polyacrylamide networks permit independent control of modulus, mesh size and ligand density, but unmodified forms are nonadhesive and require RGD-, IKVAV-, laminin- or collagen-related functionalization [42,47]. Porous membranes regulate cell–cell distance and paracrine exchange, while sacrificial materials determine lumen geometry and must be completely removed [11]. Consequently, an apparent permeability change may arise from junctional biology, membrane porosity, gel mesh size, adsorption to the device body, residual processing material or interfacial leakage; device-only, coating-only, gel-only and acellular transport controls are therefore required [70,132,146].

**Figure 5:**
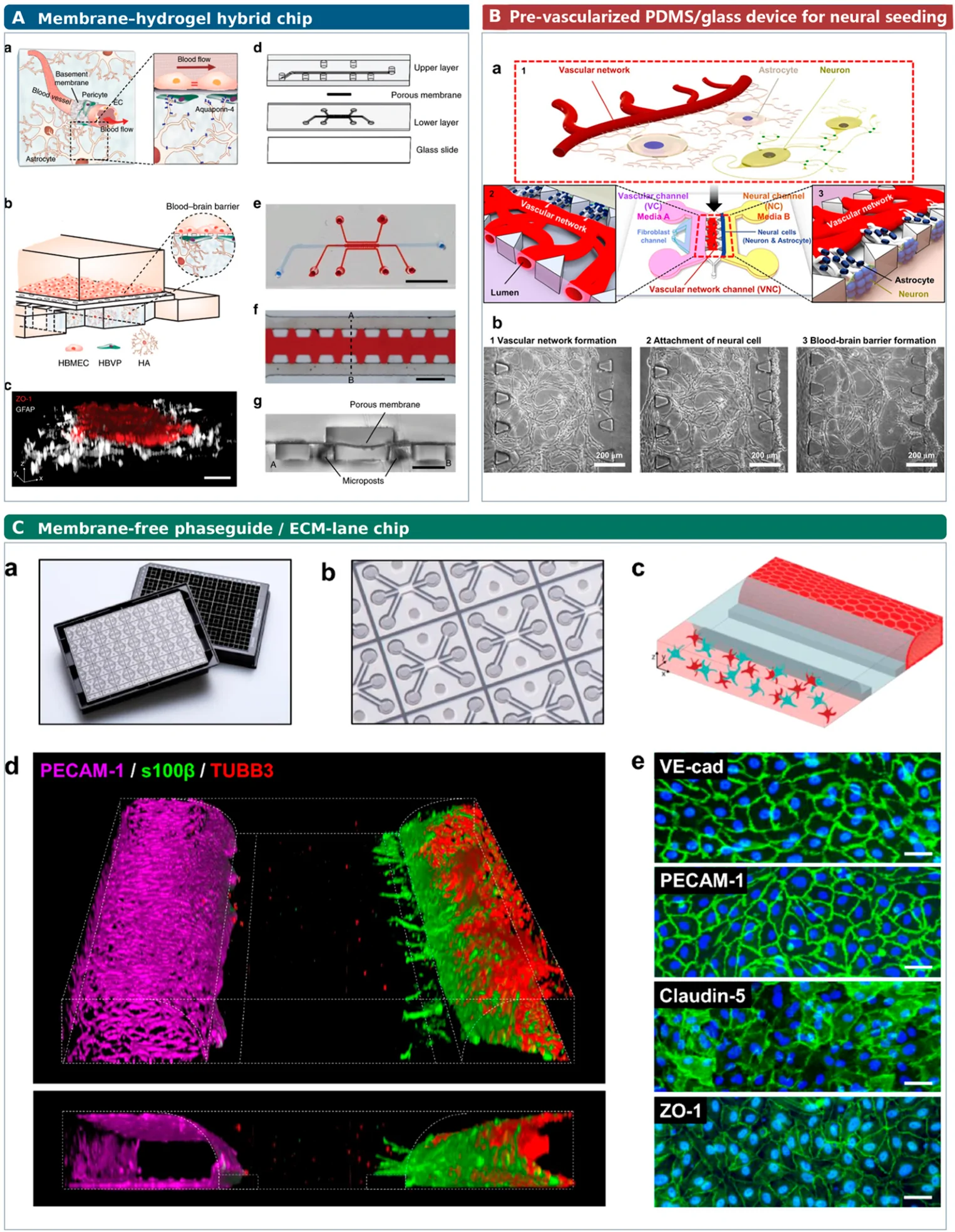
Comparison of microfluidic BBB/NVU structures and their cell–material interfaces. (**A**) A membrane–hydrogel hybrid chip with an upper endothelial channel, a porous membrane and a micropillar-confined lower Matrigel compartment, adapted from Ahn et al. [11]. (**B**) A PDMS/glass device in which endothelial cells and fibroblasts form a perfusable vascular network in fibrin before neural-cell loading, adapted from Bang et al. [131]. (**C**) A membrane-free, three-lane phaseguide platform with an endothelial vessel against collagen I gel and astrocyte/neuronal networks in the adjacent lane, adapted from Wevers et al. [132]. The source figures were cropped, resized and assembled; scientific image content was not altered. All three source articles and their figures are licensed under CC BY 4.0.

The three open-access examples in Figure 5 show how the design choice changes both fabrication and interpretation. Ahn et al. soft-lithographed two PDMS channel layers (Figure 5A), plasma-bonded them around a 7 μm PC membrane with 8 μm pores, retained 5 mg/mL Matrigel between lower-layer micropillars and perfused the endothelial channel at 16 μL/min (approximately 4 dyn/cm^2^); side channels enabled separate sampling of the perivascular gel [11]. Bang et al. bonded a soft-lithographed PDMS replica to glass (Figure 5B), injected a thrombin-crosslinked fibrin mixture containing endothelial cells and fibroblasts, allowed a perfusable network to self-assemble and subsequently loaded neurons and astrocytes on the neural side; the fibrin matrix supported VEGF-gradient-driven astrocyte migration and direct vascular contact, but the use of HUVECs and self-organized geometry limited brain specificity and reproducibility [131]. Wevers et al. used phaseguides to confine collagen I in the middle lane of 40 parallel chips (Figure 5C), coated the endothelial lane with growth-factor-reduced Matrigel, seeded astrocytes/neurons and then brain endothelial cells, and generated bidirectional perfusion on a rocking platform [132]. These are not interchangeable chip designs: membrane thickness dominates the first, fibrin remodeling and network variability dominate the second, and collagen-wall integrity plus hydrostatic rocking dominate the third.

Fabrication methods include photolithography and PDMS soft lithography, laser cutting, thermoplastic hot embossing or injection molding, plasma/thermal/solvent bonding, membrane lamination, capillary pinning between micropillars, microneedle templating, sacrificial printing, coaxial printing and cell-driven vasculogenesis [15,41]. Applicable validation methods should be matched to the architecture: TEER or impedance spectroscopy for electrode-accessible planar and lane-based models; fluorescent tracers of several molecular sizes and mass-spectrometric quantification for permeability; particle tracking or microparticle image velocimetry for flow; live confocal imaging for 3D morphology and leakage; ZO-1, claudin-5, occludin, VE-cadherin, GLUT1 and P-gp assays for phenotype; and ELISA, flow cytometry, transcriptomics or LC–MS for compartment-specific responses [56,112,113]. Oxygen/glucose deprivation, cytokines, patient-derived cells, nanoparticles and candidate drugs can be applied as controlled perturbations, but every experiment requires device-only, material-only, coating-only and flow-only controls [114,118,132].

**Table 5:** Material-by-material comparison across the five microfluidic BBB-chip categories described in Section 4.3.

Microfluidic Category	Biomaterial Examples	Function in the System	Preparation/Integration	Principal Cell Interaction	Advantages	Limitations	Representative Reference
Parallel channels	PDMS or PDMS/glass	Channel body and molded divider	SU-8 master; 10:1 PDMS casting; thermal cure; oxygen-plasma bonding	Defines flow and gas transfer; adsorbs ECM proteins before endothelial seeding	Rapid prototyping; optical transparency; gas permeability	Hydrophobic-drug sorption, oligomer leaching and thick divider.	[15,16,17,147,148,149]
	PMMA, COC or COP	Low-sorption rigid channel body	Hot embossing or injection molding; thermal, solvent or adhesive bonding	Requires surface activation and protein coating for adhesion	Scalable; lower small-molecule sorption than PDMS	Bonding residues, low gas permeability and less flexible prototyping.	[15,16,17]
	Collagen IV, fibronectin or laminin	Cell-adhesive luminal coating	Adsorption or covalent immobilization after surface activation	Integrin-mediated endothelial adhesion, spreading and junction maturation	Simple ligand presentation under controlled shear	Coating density and stability under flow can vary.	[15,147,148]
Porous-membrane co-culture	PC, PET or PTFE membrane	Separator and mechanical support	Membrane lamination between aligned channels; plasma treatment and ECM coating	Opposing endothelial and glial layers exchange signals through pores	TEER/impedance and separate luminal/abluminal sampling	Artificial stiffness/thickness, pore effects and bonding leakage.	[9,11,133,134,135,136,137,138]
	Electrospun PCL or PLGA fibers	Thin fibrous basement-membrane support	Electrospinning, collector transfer and bonding into the chip	Fiber topology and adsorbed ECM guide endothelial attachment	Higher porosity and more ECM-like architecture than track-etched films	Variable fiber/pore size, opacity and difficult lamination.	[15,16,17,42]
	Ultrathin ECM hydrogel on sparse nanofibers	Engineered basement membrane	Hydrogel deposition/polymerization on a nanofiber scaffold	Combines biochemical adhesion with reduced endothelial–glial distance	Joint control of thickness, stiffness and ECM composition	More complex fabrication and acellular permeability correction.	[42]
Hydrogel-micropillar	Collagen I	Soft 3D interface or gel wall	Neutralization on ice; injection between posts; thermal fibrillogenesis	Native adhesion, cell spreading and protease-mediated remodeling	Defined protein matrix; direct contact and confocal imaging	Contraction, weak mechanics and lot-dependent fibrils.	[45,132,146,150]
	Fibrin	Angiogenic and cell-remodelable matrix	Fibrinogen mixed with cells; thrombin-mediated gelation in the gel lane	Supports endothelial sprouting and pericyte/astrocyte contact	Rapid cell-compatible curing and vasculogenesis	Rapid degradation, compaction and variable network geometry.	[44,131]
	Matrigel	Basement-membrane-rich 3D matrix	Cold loading followed by thermal gelation and capillary pinning	Supports astrocyte networks and endothelial–perivascular signaling	Highly bioactive and easy to load	Tumor-derived, undefined and lot-dependent.	[11]
	PEG, GelMA or HA-based hybrid	Tunable synthetic/semisynthetic gel wall	Thiol–ene/Michael addition, photopolymerization or ionic/covalent co-crosslinking	Ligand density, degradability, mesh size and modulus can be programmed	Reproducible and mechanically tunable	PEG/HA require ligands; light/initiator toxicity and residual monomer.	[47,70,107]
Three-dimensional tubular vessel	Collagen I or fibrin	Structural ECM surrounding a cylindrical lumen	Gel around a needle or wire; cure and withdraw template; endothelialize lumen	Endothelium contacts ECM circumferentially under shear	Vessel-like geometry and direct abluminal matrix contact	Lumen collapse/contraction and difficult TEER.	[130,139,140,141]
	GelMA or alginate	Printable hollow or core–shell vessel wall	Photocrosslinking or Ca^2+^ ionic gelation during coaxial printing	Shape-retaining wall; GelMA provides motifs, alginate needs functionalization	Fast geometric control and print fidelity	Light/initiator exposure; alginate is nonadhesive without modification.	[24,142,143,144,145]
	Gelatin or Pluronic	Temporary sacrificial lumen template	Print template; embed in structural gel; remove thermally or by washing	Indirectly defines the endothelial lumen rather than providing long-term cues	Branched and customized channel geometry	Incomplete removal, osmotic/thermal stress and residue effects.	[130,139,140,141,142,143,144,145]
Self-assembled vascular barrier	Fibrin	Vasculogenic matrix for multicellular networks	Endothelial cells, pericytes/astrocytes and thrombin-crosslinked fibrin cultured under perfusion	Cell-driven sprouting, anastomosis and direct multicellular contact	Capillary-scale perfusable networks	Irregular diameter and connectivity.	[44,131]
	Collagen I	Fibrous matrix or phaseguide-confined ECM wall	Neutralized collagen injected and thermally gelled before/with cell loading	Integrin-mediated adhesion and contact guidance	Familiar, remodelable matrix; compatible with lane-based chips	Contraction and fiber variability.	[132]
	Fibrin plus collagen I microfibers	Hybrid matrix with biochemical and topographical cues	Disperse collagen microfibers in cell-laden fibrinogen; add thrombin and gel	Microfibers improve astrocyte survival and guide capillary-network maturation	Reduced randomness and increased network formation	Fiber dose/orientation and hybrid-gel homogeneity require control.	[46]

#### 4.3.1. Parallel Microchannel-Separated Barrier Models

**Figure 6:**
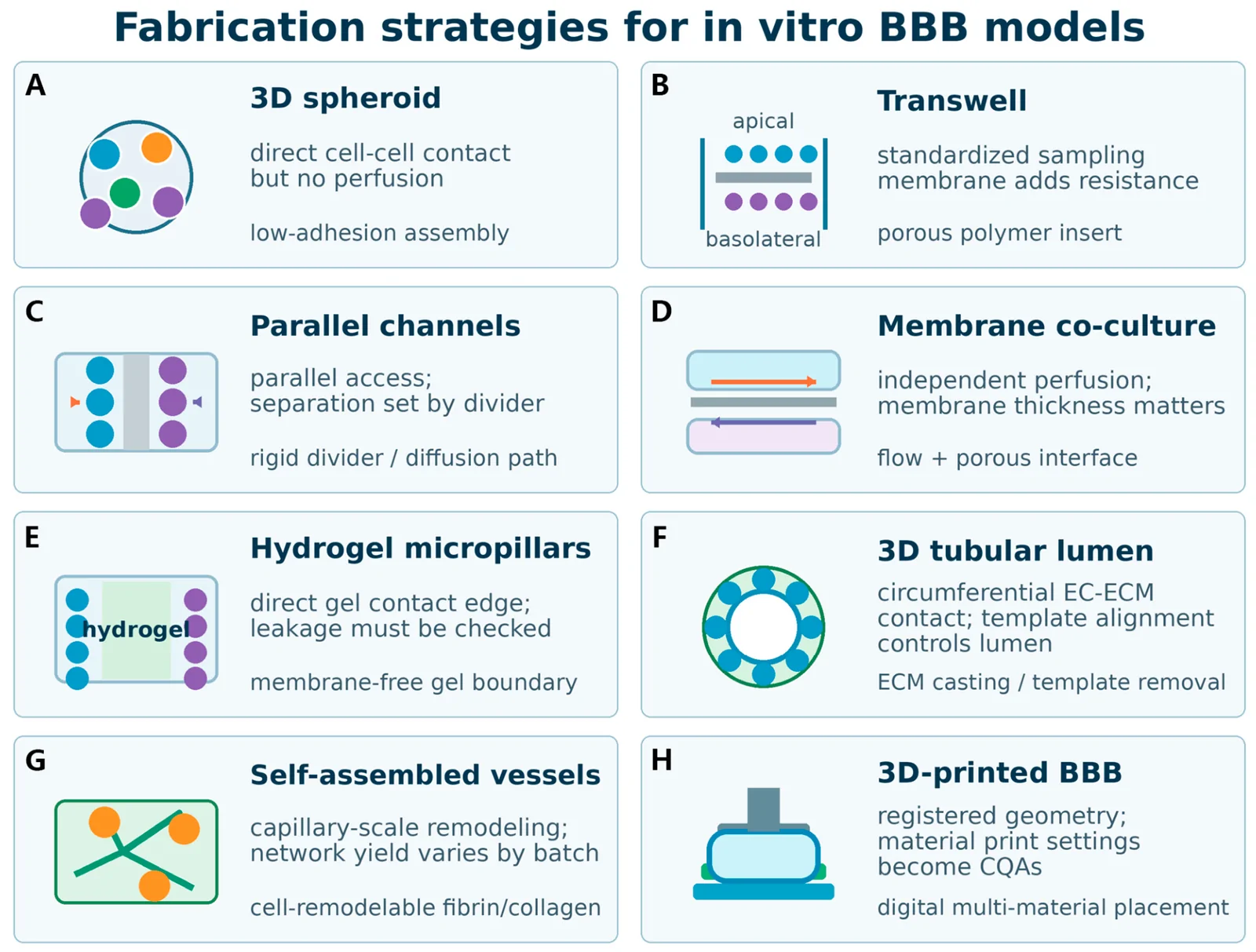
Comparison of fabrication strategies for in vitro BBB models. (**A**) Three-dimensional spheroid model [119]; (**B**) Transwell platform [126]; (**C**) parallel microchannel-separated model; (**D**) porous-membrane co-culture model [9]; (**E**) hydrogel-micropillar model [146]; (**F**) three-dimensional tubular model [139]; (**G**) self-assembled cellular barrier model [44]; and (**H**) 3D-printed BBB model [22]. Created by the authors based on the platform geometries reported in the cited studies; no third-party graphical elements were reproduced.

Parallel-channel models place vascular and neural channels side by side, separated by microslits or posts (Figure 6C). Deosarkar et al. fabricated the neonatal B3C as an optically clear PDMS chip with two vascular channels surrounding a tissue compartment; perfusion improved barrier characteristics relative to a static Transwell membrane and produced permeability comparable to the neonatal-rat reference used in that study [147]. Prabhakarpandian et al. likewise used the side-by-side PDMS SyM-BBB to expose endothelial cells to flow while supplying astrocyte-conditioned medium through the adjacent compartment, reporting increased tight-junction expression and a restricted barrier [148]. These studies explain PDMS’s popularity: soft lithography reproduces micrometer features accurately, the elastomer is transparent and gas permeable, plasma treatment permits irreversible bonding to glass, and the low-cost workflow supports rapid design iteration and live imaging [15,148].

PDMS also introduces test-specific bias. Van Meer et al. quantified the loss of four drugs from PDMS-coated wells by HPLC and showed that absorption was compound-dependent, time-dependent and altered by coatings and cells; a nominal inlet concentration can therefore differ from the dose reaching a BBB channel [149]. Additional disadvantages reported for PDMS microphysiological systems include nonspecific protein adsorption, leaching of uncrosslinked oligomers, evaporation through the elastomer, transient hydrophilicity after plasma oxidation and poor control of dissolved gases. Moreover, a molded PDMS divider is much thicker and stiffer than the native basement membrane [15,149]. These effects require device-only recovery experiments, extraction controls and measurement of inlet/outlet drug concentration rather than assuming inert transport.

PDMS is not the only device material. Glass has low sorption, stable optics and chemical resistance; Ahn et al. used a PDMS/glass hybrid construction, and Booth and Kim combined four PDMS substrates with two glass layers around a porous PC membrane, demonstrating that rigid glass can supply optical and structural support while PDMS supplies patterned channels [11,135]. Fully rigid PMMA, cyclic olefin copolymer (COC) and cyclic olefin polymer (COP) can be laser-machined, hot-embossed or injection-molded and generally show lower hydrophobic-compound sorption than PDMS, making them attractive for pharmacokinetic screening and scale-up [17]. Their disadvantages are less flexible prototyping, low gas permeability and the need to control thermal-, solvent- or adhesive-bonding residues. Regardless of body material, collagen IV, fibronectin or laminin coating is normally required for endothelial adhesion, and ligand density and retention under flow must be measured [15,147,148].

#### 4.3.2. Porous-Membrane Co-Culture Barrier Models

Porous-membrane chips laminate PC, PET, PTFE or an electrospun polymer interface between aligned channels (Figure 6D). Ahn et al. plasma-bonded PDMS layers around a 7 μm PC membrane with 8 μm pores and placed Matrigel below the membrane, thereby combining a mechanically defined separator with a three-dimensional perivascular matrix [11]. Booth and Kim used a porous PC membrane in a multilayer PDMS/glass μBBB and integrated TEER and permeability measurements [135]. Sellgren et al. replaced the commonly used polyester film with a hydrophilized, optically transparent nanoporous PTFE membrane; endothelial cells tolerated physiological shear while astrocytes were maintained in a 3D hydrogel, improving optical access to the co-culture [138]. These examples show that PC offers standardized geometry, PTFE improves chemical resistance and imaging after hydrophilization, and the device body may be a hybrid rather than a single polymer [11,135,138].

A thinner ECM-mimetic interface can reduce the cell–cell distance. Choi et al. supported an ultrathin ECM-hydrogel engineered basement membrane with a sparse electrospun nanofiber scaffold; the approximately 500 kPa interface supported iPSC-derived brain endothelial cells with strong physical-barrier and efflux-pump functions [42]. The gain in biological relevance comes with greater process sensitivity: nanofiber diameter, pore distribution, hydrogel thickness and transfer/bonding must be reproduced [15,16]. More generally, membrane pore density changes acellular permeability, thickness delays paracrine exchange, coating modifies adhesion, trapped bubbles or incomplete bonding cause leakage, and cells can migrate through large pores [9,17]. Membrane polymer, thickness, pore size, porosity, coating mass and acellular transport should therefore accompany TEER or cellular permeability data [42,138].

#### 4.3.3. Hydrogel-Micropillar Barrier Models

Micropillar devices use capillary pinning between posts to retain a cell-laden hydrogel beside a perfusion channel (Figure 6E). The gel replaces the rigid membrane, enabling direct endothelial contact with a soft 3D matrix and short-range interaction with astrocytes or pericytes. Adriani et al. confined collagen I gels containing neurons and astrocytes in adjacent lanes and cultured cerebral endothelial cells along the gel interface, creating a membrane-free neurovascular model for imaging cell–cell interaction [146]. Ahn et al. instead retained Matrigel between lower-channel micropillars beneath a PC membrane, showing that micropillar confinement can also be combined with a membrane when separate luminal and abluminal sampling is required [11]. DePalma et al. used a brain-mimetic, IKVAV-containing HA/gelatin-based hydrogel in a 3D microfluidic BBB model and showed that glioblastoma cells altered permeability and endothelial immune-cell adhesion [107]. For material comparison, the following discussion is divided explicitly into natural-polymer and synthetic-polymer hydrogels. In both groups, post gap, precursor viscosity and gelation kinetics control leakage, whereas ligand identity, modulus and degradability control cell behavior [41,107,146].

##### Natural-Polymer Hydrogels

Natural-polymer hydrogels are formed from ECM-derived or biologically sourced macromolecules, including collagen I, fibrin, gelatin, hyaluronic acid and basement-membrane extracts, such as Matrigel. In Adriani et al., neutralized collagen I was thermally fibrillized in the chip and supplied a remodelable matrix for neurons and astrocytes next to the endothelial lane [146]. Campisi et al. and Bang et al. used thrombin-crosslinked fibrin to support self-organized perfusable vessels and direct contact with perivascular cells [44,131], whereas Ahn et al. used cold-loaded, thermally gelled Matrigel as the perivascular phase [11]. Advantages of these materials include intrinsic cell-adhesion motifs, enzyme-mediated remodeling, cytocompatible gelation and strong support for angiogenesis or direct endothelial–glial interaction. Their disadvantages are weaker control of composition and mechanics: collagen fiber architecture changes with pH, temperature and concentration, and the gel can contract; fibrin compacts and degrades rapidly; and Matrigel is tumor-derived, compositionally undefined and lot-dependent [44]. Natural matrices are therefore biologically instructive but require lot qualification, acellular permeability controls and measurement of modulus, contraction and degradation [45,131,146].

##### Synthetic-Polymer Hydrogels

Synthetic-polymer hydrogels are assembled from chemically defined precursors such as PEG, PVA and polyacrylamide; engineered semisynthetic derivatives such as GelMA and methacrylated HA are included here as bridging materials because their crosslink density is similarly programmed during fabrication [47,70,105]. Ahmad et al. covalently incorporated biomimetic peptides into a PEG network and co-cultured endothelial cells with astrocytes, demonstrating that ligand identity can be specified instead of inherited from an undefined matrix [70]. Augustine et al. used photocrosslinked GelMA to construct a BBB model for breast-cancer brain metastasis [47], while DePalma et al. combined HA/gelatin chemistry with an IKVAV-bearing component in a microfluidic model [107]. Advantages include reproducible composition, independent control of modulus and mesh size, programmable degradability, and covalent incorporation of adhesion peptides. Disadvantages include the poor intrinsic adhesiveness of unmodified PEG, PVA and HA, possible restriction of diffusion or cell spreading at high crosslink density, and toxicity from residual monomer, photoinitiator or excessive light exposure. Synthetic systems therefore require ligand-free, matched-modulus and matched-ligand controls to separate chemical signaling from mechanics [105,107].

#### 4.3.4. Three-Dimensional Tubular Barrier Models

Tubular systems create a cylindrical lumen within an ECM (Figure 6F). Kim et al. positioned microneedles in a 3D-printed frame, gelled collagen I around them and withdrew the templates to obtain parallel microchannels that were subsequently endothelialized [139]. Partyka et al. used an endothelialized lumen in a compliant collagen matrix and showed that flow-derived mechanical stress regulated both barrier permeability and solute transport along the surrounding matrix [141]. These examples retain direct circumferential endothelial–ECM contact but depend on collagen gelation, template alignment and withdrawal without tearing. Alternative methods print sacrificial gelatin or Pluronic, embed it in a structural gel and remove it, or use coaxial extrusion to form hollow alginate, GelMA or composite filaments directly [130,139,140,141,142,143,144,145].

Wang et al. recently used coaxial extrusion to fabricate a three-layer vascular construct with BBB function; the printed barrier could be disrupted by hypertonic mannitol and subsequently recover, providing a material-processing example for neuroprotective-drug screening [24]. Printing improves geometric repeatability and multilayer placement, but the constituent polymers have different roles: GelMA contributes cell-adhesive motifs and photocrosslinked shape retention, alginate provides rapid ionic gelation but is nonadhesive unless modified, and sacrificial inks must be completely removed without osmotic or thermal injury [24,142,143,144,145]. Collagen and fibrin are more readily remodeled but can contract or deform under perfusion. Tubular geometry is physiologically attractive, yet TEER electrode placement and recovery of abluminal samples remain difficult; studies commonly infer permeability by imaging tracer leakage and must correct for gel diffusion and lumen diameter [24,139,141].

#### 4.3.5. Self-Assembled Vascular Barrier Models

Self-assembled chips load vascular and perivascular cells into a fibrin or collagen compartment and rely on vasculogenesis and angiogenesis to create perfusable networks (Figure 6G). Earlier microvasculature-on-a-chip and endothelial-sprouting studies established how long-term perfusion, obstruction and fluid forces alter vascular organization, providing process controls for interpreting BBB-specific self-assembly [151,152]. Campisi et al. embedded human endothelial cells, pericytes and astrocytes in fibrin and obtained a perfusable three-cell BBB microvascular network [44]. Bang et al. also used thrombin-crosslinked fibrin to form a perfusable vascular network that directly contacted astrocytes after neural-cell loading [131]. In a more recent material intervention, Nakayama-Kitamura et al. dispersed collagen I microfibers in fibrin; the fibers improved astrocyte survival and accelerated capillary-network formation and maturation through an integrin-β1-related mechanism [46]. Every example therefore links a specific matrix choice to vessel assembly rather than treating self-organization as a cell-only process [44,46,131].

The major advantages are capillary-scale geometry, direct multicellular contact and cell-driven ECM remodeling. The drawbacks documented across these systems are irregular lumen diameter and connectivity, batch-dependent perfusable-network yield, fibrin compaction/degradation and limited compatibility with TEER [44,46,131]. Collagen microfibers add topographical guidance but introduce new quality-control variables, including fiber length, diameter, dose, orientation and dispersion [46]. Material improvements should therefore target defined fibrin degradation, reproducible fiber architecture and quantitative perfusion yield; permeability should be reported together with network morphology and acellular gel transport rather than as a single averaged value [12,41,46].

### 4.4. BBB-Specific 3D Printing: Materials and Fabrication

**Figure 7:**
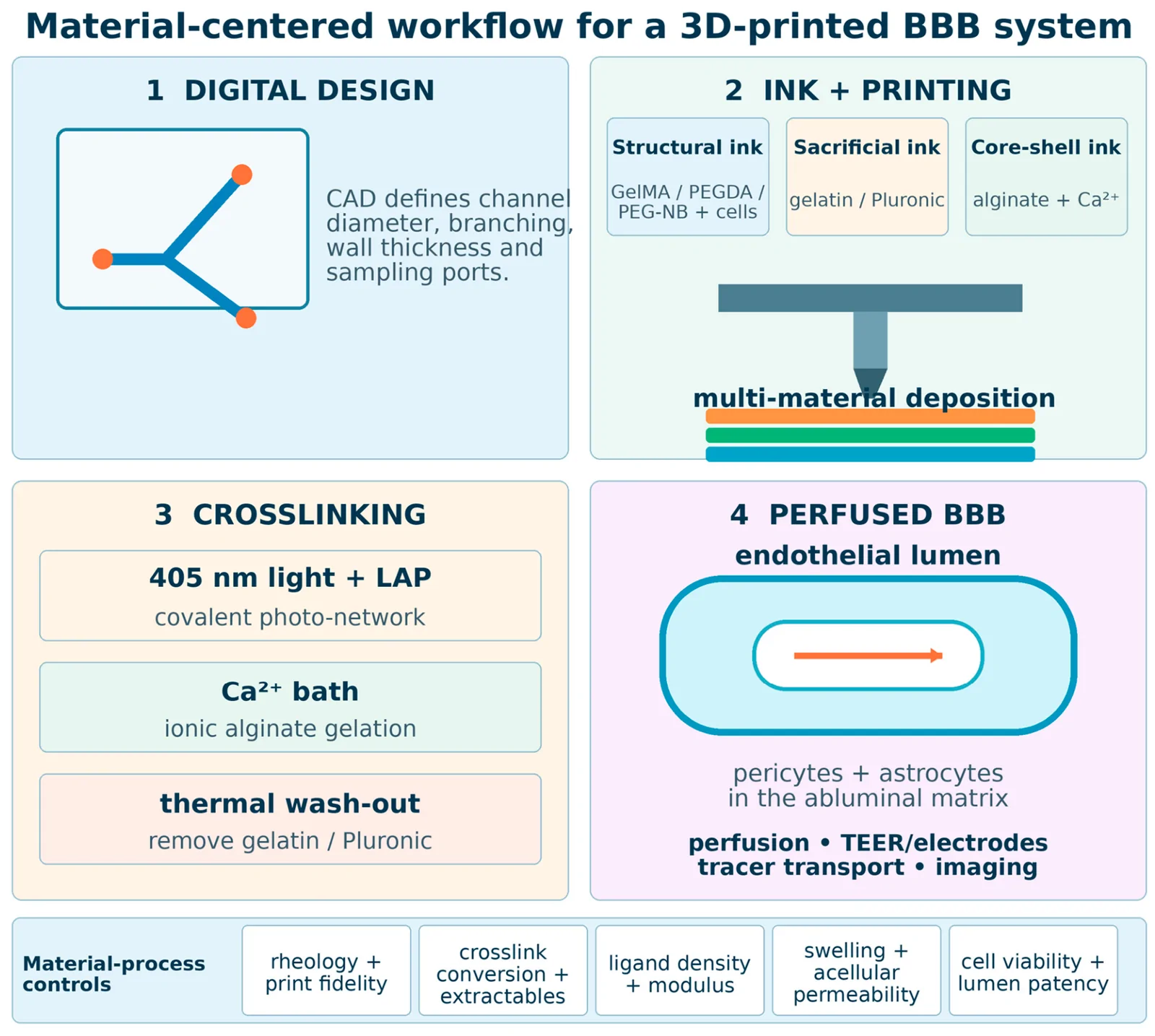
Material-centered schematic of a 3D-printed BBB system. A digital vascular design is translated through multi-material deposition, followed by chemistry-specific crosslinking and removal of any sacrificial ink. The printed lumen is subsequently endothelialized and perfused while pericytes and astrocytes are placed in the abluminal matrix. Original schematic created for this review; no third-party graphical elements were reproduced.

Three-dimensional printing is not a single BBB model but a family of additive manufacturing routes that translate a digital vascular geometry into a rigid device, a sacrificial channel template or a cell-laden hydrogel construct [19,25]. For biomaterials analysis, it is essential to distinguish cell-free printing of molds and housings from bioprinting, in which cells and matrix precursors are deposited together [22]. The BBB-relevant process classes are extrusion and coaxial extrusion, vat photopolymerization by digital light processing (DLP) or stereolithography, and indirect printing with a removable sacrificial or support ink [25,26,27]. Research from vascular, hollow-organ and perfusable-tubule printing defines the material requirements for hollow-channel fidelity, sacrificial-ink removal and sustained perfusion even when the printed tissue is not itself a BBB model [153,154,155]. Figure 6H places 3D printing within the wider landscape of BBB fabrication, whereas Figure 7 isolates the material operations that determine printing fidelity and biological performance [27,104,156].

The printable formulation must satisfy competing requirements before, during and after deposition [19,25]. Extrusion accepts collagen, fibrin, GelMA, alginate and composite inks over a broad viscosity range, but viscosity that is high enough to retain a filament also increases nozzle pressure and cell shear [22,26]. Coaxial nozzles generate hollow or multilayer filaments and can combine rapid Ca^2+^-mediated alginate gelation with a more cell-adhesive phase, although concentric flow-rate mismatch changes wall thickness [24,157]. DLP and stereolithography provide nozzle-free, layerwise patterning of PEGDA, PEG-norbornene (PEG-NB) or GelMA with a photoinitiator; their resolution depends on light absorption, conversion and swelling, while unreacted precursor, radical dose and optical attenuation remain material-specific risks [142,143,144,145,158]. Sacrificial gelatin or Pluronic templates can generate perfusable lumens inside collagen, fibrin or photocrosslinked hydrogels, but template removal, interface bonding and acellular permeability must be verified [26,159,160,161].

Recent light-based studies show why composition must be discussed together with printer settings. Galpayage Dona et al. used DLP to fabricate a perfusable branching scaffold from PEGDA and 15% GelMA, selected after stiffer PEGDA formulations reduced optical access and promoted endothelial clumping. The formulation combined PEGDA-derived structural stability with gelatin-derived adhesion, while collagen I, heparin-containing Heprasil and Matrigel were evaluated as additional cell-attachment phases; astrocytes were embedded around lumens that were seeded with endothelial cells and pericytes [162]. Paone et al. instead printed a PEG-NB network and spatially clicked RGD, IKVAV or HAVDI peptides into either the channel wall or bulk gel. HAVDI- and IKVAV-lined channels increased endothelial coverage and junctional ZO-1, whereas RGD in the surrounding matrix supported endothelial–astrocyte co-culture, demonstrating that DLP can pattern biochemical identity as well as topology [95].

Royse et al. used projection stereolithography to print a scalable perfusable BBB construct from 10 wt% GelMA, 3.25 wt% low-molecular-weight PEGDA, LAP photoinitiator, tartrazine photoabsorber and glycerol. The hybrid bioink balanced cell adhesion, mechanical support, optical resolution and viscosity; astrocytes were embedded in the matrix, and a 700 μm channel was subsequently populated with pericytes and endothelial cells [163]. The formulation enabled perfusion and barrier/transport analyses, but its several functional additives also illustrate a validation burden: light dose, conversion, leachable photoabsorber, swelling and the transport resistance of the acellular printed wall must be measured independently before biological permeability is interpreted [163].

Extrusion-based work provides a complementary material strategy. Wang et al. used a collagen-based low-viscosity composite reinforced with alginate to bioprint neurovascular tissue, exploiting collagen bioactivity while using the second polymer to improve shape retention and printing continuity [164]. More recently, Wang et al. applied coaxial deposition to generate a three-layer vessel containing endothelial cells, pericytes and astrocytes and used the construct for neuroprotective-drug screening [24]. Coaxial organization improves radial placement and permits rapid stabilization of a hollow filament, but lumen diameter, layer mixing, interfacial adhesion and sequential crosslinking must be reported because these variables alter wall diffusion and shear. These studies therefore support 3D printing as an emerging BBB route while showing that a printed geometry is only meaningful when the material history is quantitatively defined [24,164]. Recent 3D-printed BBB and neurovascular systems, are summarized in Table 6.

Across these studies, no material can be ranked independently of the process. PEGDA and PEG-NB offer defined photochemistry and geometric precision but require biofunctionalization; GelMA and collagen improve adhesion and remodeling but introduce concentration-, source- and temperature-dependent rheology; alginate stabilizes extruded structures rapidly but lacks intrinsic mammalian-cell adhesion; sacrificial gelatin or Pluronic improves lumen formation but creates a removal and extractables problem [26,104,156]. A rigorous comparison should therefore report pre-print viscosity and yield stress, nozzle diameter or projected pixel/layer thickness, pressure or light dose, initiator and absorber concentration, crosslink conversion, swelling, modulus, ligand density, cell viability, lumen patency and acellular permeability [162,163,164].

The field remains emergent rather than mature. Most printed BBB channels are still much larger than brain capillaries, TEER is difficult to integrate in cylindrical matrices, and published studies use different cells, geometries, flow rates and tracer sizes [24,25,95]. Multicomponent inks also make causal attribution difficult because print fidelity, stiffness, ligand presentation and diffusion change simultaneously [24,162,163]. The next step is not simply greater anatomical complexity, but factorial material studies under matched geometry and shear, including cell-free transport controls, standardized photocrosslinking or ioncrosslinking records, low-sorption manifolds, automated perfusion and long-term assessment of junctions, transporters and compound recovery [24,164].

### 4.5. Analytical Techniques for Evaluating BBB Integrity

#### 4.5.1. Transendothelial Electrical Resistance

Cerebral vascular beds exhibit far tighter barrier properties than vasculature in peripheral organs. Tight junction proteins within brain endothelial monolayers restrict the flux of small ions such as Na^+^ and Cl^−^, generating quantifiable transendothelial electrical resistance (TEER) across the cell layer [38]. For TEER quantification, one electrode is placed in the vascular compartment and the second in the brain-mimicking compartment. Butt et al. reported in vivo TEER values as high as 5900 Ω·cm^2^ in mouse cerebral vasculature [165,166].

Primary cell-derived in vitro BBB models yield TEER values up to 600 Ω·cm^2^ for rat brain microvascular endothelial cells (BMECs) and 1800 Ω·cm^2^ for bovine primary BMECs, respectively [166]. Furthermore, human induced pluripotent stem cell (iPSC)-derived endothelial cells generate physiological TEER values ranging from 4000 to 5000 Ω·cm^2^ in vitro [23,167]. These results highlight their superior barrier phenotypes compared with immortalized human brain endothelial cell lines (e.g., hCMEC/D3, with TEER ranging from 50 to 1200 Ω·cm^2^) [168]. Nevertheless, immortalized human brain microvascular endothelial cells remain the most widely adopted cell source in this research field, due to critical limitations of primary human brain endothelial cells: scarce tissue sources, high risk of contamination during isolation, limited cell yield, excessive costs, unstable barrier phenotypes, as well as complicated, time-consuming, costly and error-prone workflows for endothelial differentiation from stem cells [169,170].

Although TEER measurement is regarded as the gold standard for quantifying BBB tightness, multiple μBBB platforms are incompatible with this detection method due to structural design constraints, including hydrogel micropillar barrier models, 3D tubular vessel models, and self-assembled vascular μBBB systems [130,150,166].

#### 4.5.2. Permeability Assays

A second mainstream approach to assess BBB integrity is small-molecule permeability testing, which quantifies the diffusion rate of molecular tracers across the barrier membrane. Tracers must be carefully selected to avoid disrupting native BBB physiological function [171,172,173]. Fluorescently labeled dextrans are the most commonly used probes: they readily permeate a compromised BBB while being efficiently excluded by intact, functional barriers. In general, physicochemical properties of tracers, including molecular size and polarity, govern their BBB penetrance. Additionally, low-molecular-weight fluorescent dyes such as sodium fluorescein (376 Da) and Cascade Blue™ (530 Da) have been validated as tracers for BBB integrity evaluation [9,174].

## 5. Material Design Principles and Preparation Technologies for In Vitro BBB Models

BBB models are built to answer transport, toxicity, inflammation, tumor-invasion or neurovascular-coupling questions. The medical requirement can therefore be stated briefly: the construct must maintain a selective endothelial barrier while exposing the vascular–glial interaction relevant to the assay. The remainder of this chapter focuses on how material chemistry, processing and device interfaces satisfy that requirement [12,15,112].

### 5.1. Biomaterial Design Principles and Comparative Analysis

Five coupled principles guide material selection. First, the luminal surface must support endothelial adhesion, polarity and shear without absorbing the test compound. Second, the abluminal matrix should present defined collagen-, laminin- or hyaluronan-related cues at brain-relevant compliance. Third, mesh size, swelling and degradation must permit nutrients and signaling while contributing a measurable, cell-independent resistance to tracer transport. Fourth, curing or printing must preserve cells and produce reproducible geometry. Fifth, the material and analytical interface—electrodes, optical windows, sampling ports and bonds—must remain stable for the full experiment [12,15,16,17,173,175]. These criteria require reporting composition, functionalization, crosslink density, modulus, swelling, degradation and acellular permeability rather than describing chip architecture alone.

Comparative studies show why no single hydrogel is universally optimal. Fibrin enabled Campisi et al. to form perfusable endothelial–pericyte–astrocyte networks, but self-assembled geometry and yield remained variable [44]. Collagen I supported a stem-cell-derived hydrogel BBB and allowed remodeling, yet contraction, diffusion lag and fibril variability required control [45]. Photocrosslinked GelMA improved patternability and cell adhesion but introduced methacrylation-, initiator- and light-dose variables [47]. Peptide-functionalized PEG separated ligand identity from network mechanics: Ahmad et al. built a defined endothelial–astrocyte matrix, and Paone et al. spatially assigned RGD, IKVAV and HAVDI within a printed PEG-NB construct [70,95]. Nakayama-Kitamura et al. added collagen I microfibers to fibrin and improved capillary-network formation, whereas Choi et al. used an ultrathin engineered basement membrane to enhance an iPSC-derived barrier [42,46]. These interventions are informative because each changes a specific material feature—ligand, topology, fiber guidance or interface thickness—rather than merely adding more cell types.

The principal gap is comparability. Natural, synthetic and hybrid matrices are usually tested with different endothelial sources, flow rates, dimensions and readouts, so their reported TEER or permeability values cannot be treated as a material ranking. The meta-analysis by Shamul et al. documented substantial heterogeneity in the composition and reported properties of in vitro BBB models [12]. A valid comparison should therefore use the same cell batch and shear, match modulus or ligand density where appropriate, include empty-device and acellular-gel transport controls, and measure TEER, several tracer sizes, junctional markers, transporter activity and compound recovery [12,42,44,45,46,47,70].

Recent organ-on-a-chip literature is converging on six topics: engineered ultrathin basement membranes; iPSC- or patient-derived cells; integrated TEER and optical sensing; vascularized organoids and tumor interfaces; higher-throughput perfusion arrays; and low-sorption thermoplastics that can replace PDMS for compound screening [12,15,16,17,107]. For example, DePalma et al. used an IKVAV-containing HA/gelatin matrix to study glioblastoma-induced barrier changes, while recent printed BBB systems combined PEGDA/GelMA or peptide-patterned PEG-NB with perfusable lumens [95,162,163]. These advances remain material problems: thin interfaces must resist tearing, sensors must be bonded without leakage, organoid matrices must vascularize without uncontrolled contraction, and scalable devices must reproduce surface chemistry. The chip should therefore be treated as a controlled boundary condition around the biomaterial rather than the organizing subject of the chapter [46,162]. Material-centered comparison of representative BBB/NVU studies. are summarized in Table 7.

### 5.2. Preparation and Fabrication of Materials for BBB Applications

Preparation must be described as a chemical and manufacturing sequence rather than by polymer name alone [12,104]. Table 8 summarizes the natural, semisynthetic, synthetic, decellularized, composite, sacrificial and device materials used across the models reviewed above. For every route, precursor source and concentration, functionalization, purification, crosslinker or catalyst, curing conditions, washing and sterilization determine the final cell-contacting interface [15,16,17,105].

Two synthesis examples illustrate why processing history matters. In a GelMA route, gelatin amines are methacrylated, the polymer is dialyzed and lyophilized, and the purified product is redissolved with LAP before controlled light exposure; incomplete purification or excessive dose can change viability and permeability [47,105]. In a peptide-programmed PEG-NB route, multi-arm PEG-NB is mixed at defined thiol:ene stoichiometry with a dithiol or degradable peptide crosslinker, while cysteine-bearing RGD, IKVAV or HAVDI is added before DLP curing; Paone et al. used this orthogonality to assign different ligands to the channel wall and bulk gel [70,95]. These examples should be reported with degree of substitution, conversion and swelling, not only nominal polymer concentration.

Device manufacture is part of the same material workflow. PDMS is replica molded, thermally cured and plasma bonded, whereas glass is etched or micromachined and PMMA or COC/COP is hot-embossed or injection-molded before thermal, solvent or adhesive bonding [15,16,17]. The hydrogel is then introduced by casting, capillary filling, micropillar pinning, templating or direct printing. Before cells are added, the complete device should be tested for extractables, compound sorption and recovery, acellular permeability, bond leakage, hydrogel swelling, channel deformation and sterilization-induced surface changes. This sequence keeps Section 5 focused on materials and preparation while treating the chip only as the boundary that constrains those materials.

A practical method choice also requires operational criteria that are rarely compared in the same experiment. Table 9 therefore provides a qualitative decision matrix derived from the comparative and review reports in Section 3, Section 4 and Section 5. “Reproducibility” combines material-batch and geometric variation; “scale/HTS” denotes compatibility with parallel manufacture and plate-based automation; “long-term stability” includes material geometry and sustained BBB readouts; and “standardization” denotes the ease of transferring an SOP and acceptance criteria between laboratories. The high/medium/low ratings are based on relative syntheses of findings, not meta-analytic performance scores, because cell source, flow, dimensions and endpoints differ across studies [12,17].

The matrix makes the selection logic explicit. For early permeability or toxicity ranking, a standardized insert or molded thermoplastic device may be more informative than a biologically elaborate construct because replicate number, compound recovery and acceptance criteria dominate [12,15]. For mechanobiology, tumor invasion or vascular morphogenesis, hydrogel-micropillar, tubular or self-assembled systems justify lower throughput by providing direct cell–matrix contact and three-dimensional remodeling [16,42]. DLP is favored when reproducible geometry and spatial chemistry are the primary variables; extrusion or coaxial printing is favored when multilayer cell placement and material diversity outweigh resolution [162,163]. Industrial translation will most likely use hybrid platforms that combine a low-sorption scalable body, a thin defined interface, standardized cell sources and integrated sensing. Across all formats, apparent long-term stability should be demonstrated by time-resolved TEER or permeability together with junctions, transporters, geometry and compound recovery rather than by viability alone [42,163].

## 6. Clinical Translation Enabled by Biomaterials and Advanced Manufacturing

Clinical translation does not follow automatically from increasing model complexity. A BBB platform becomes decision-relevant only when its material composition, geometry and cell source are matched to a defined context of use, such as ranking CNS exposure, identifying a patient-specific barrier phenotype, selecting a therapy or explaining a disease mechanism. Biomaterials establish the cell-contacting biochemical and mechanical boundary, whereas manufacturing determines whether that boundary can be reproduced across wells, batches and laboratories. The recent innovations below should therefore be judged by both biological fidelity and their ability to deliver traceable, timely and clinically concordant measurements [47,105].

### 6.1. Precision Medicine and Patient-Derived iPSC BBB Models

Patient-derived iPSCs provide a route from an individual genotype to matched endothelial, neural and glial compartments. Vatine et al. combined patient-specific iPSC-derived brain microvascular endothelial-like cells with an organ-on-a-chip interface and demonstrated disease-related transport abnormalities in cells from patients with monocarboxylate transporter 8 deficiency, establishing an important proof of concept for personalized BBB phenotyping [176]. Park et al. used developmentally inspired hypoxic differentiation, an ECM-coated microfluidic interface, pericytes, astrocytes and physiological flow to obtain stable restriction and functional efflux and transcytosis for up to two weeks [177]. Together, these studies show how controlled oxygen history, surface ligands and shear can reveal phenotypes that static culture may miss.

The translational advantage is nevertheless conditional. iPSC lines retain donor genetics, but differentiation efficiency, endothelial identity and maturation vary between clones and batches; an apparent patient effect can therefore be confounded by cell manufacture [12,42]. Undefined basement-membrane extracts obscure causal material effects, and PDMS can absorb hydrophobic drugs. Precision-medicine studies should consequently use multiple clones, isogenic controls where possible, predefined release criteria, low-sorption device bodies and chemically defined matrices whose ligand density, modulus and thickness are qualified before cells are introduced. Without these controls, personalization increases biological variance faster than it increases predictive value [15,176,177].

### 6.2. Neurodegenerative Disease and Brain-Cancer Applications

Disease models illustrate why the required material environment changes with the clinical question. In Parkinson’s disease, Pediaditakis et al. cultured dopaminergic neurons, astrocytes, microglia, pericytes and brain endothelium under flow and reproduced alpha-synuclein-associated neuroinflammation and barrier disruption [178]. De Rus Jacquet et al. then combined patient-derived LRRK2 G2019S astrocytes with a three-dimensional microfluidic BBB model; the mutant astrocytes impaired capillary formation, whereas MEK1/2 inhibition attenuated inflammation and rescued barrier formation [179]. These platforms are useful for target validation because perfusion and direct cell–matrix contact permit vascular and neural phenotypes to be tracked together, but they remain vulnerable to donor, hydrogel and network-geometry variation.

For Alzheimer’s disease, Pavlou et al. used communicating hydrogel compartments to combine a perfusable self-assembled human microvasculature with stem-cell-derived neural cells carrying familial Alzheimer’s disease mutations. The four-week culture captured time-dependent permeability, vascular-marker changes and amyloid-beta accumulation [180]. This long duration is clinically relevant for progressive phenotypes, yet the mixed use of primary vascular cells and mutation-bearing neural cells limits attribution of a fully patient-specific response. Defined or separately tunable vascular and neural matrices would allow genotype, mechanics and matrix composition to be varied independently rather than changing together.

Brain-cancer models require an additional compromise between barrier function and a permissive tumor matrix. Earlier microfluidic blood–tumor barrier work showed that tumor-conditioned interfaces can alter permeability and therefore should not be treated as an intact-BBB control [181]. Yi et al. bioprinted patient-derived glioblastoma cells and endothelial cells in a brain decellularized-ECM bioink with a spatial oxygen gradient and reproduced patient-specific resistance to chemoradiotherapy [182]. Koh and Hagiwara subsequently created a modular BBB-in-a-CUBE that could be matured separately, transferred into a fluidic circuit and coupled to a glioblastoma tissue to test barrier permeation and downstream treatment response [183]. Bioprinting and modular assembly can preserve scarce samples and decouple tissue maturation schedules, but clinical turnaround time, dECM lot variability, print resolution, tumor sampling bias and prospective agreement with patient outcomes must be established before treatment selection can be claimed.

### 6.3. High-Throughput and AI-Assisted Drug Discovery

For pharmaceutical screening, the most valuable manufacturing advances are those that convert BBB models into analyzable data factories: molded low-sorption thermoplastics, plate-compatible fluidics, standardized thin interfaces, automated liquid handling, barcoded material lots and integrated electrical or optical sensing [15,17]. These features support concentration–response experiments, mass-balance measurements and repeated imaging at a scale that handcrafted PDMS devices or variable self-assembled networks rarely achieve [16]. The clinical benefit is not merely higher throughput; it is the ability to compare patient or disease groups against reference compounds using the same geometry and acceptance criteria [105,177].

Artificial intelligence can then integrate time-resolved permeability, morphology, junction continuity, transporter activity and multi-omics data to classify phenotypes or prioritize compounds. Rosa et al., for example, used explainable machine-learning models to identify molecular substructures associated with passive BBB penetration [184]. However, most current BBB permeability algorithms are trained on heterogeneous historical data rather than prospectively standardized human BBB-chip measurements, and published organ-on-a-chip/AI work remains dominated by workflow proposals rather than clinical validation [184,185]. A stronger strategy is a closed experimental loop in which an interpretable model proposes compounds, a standardized BBB platform generates new transport and toxicity labels, and active learning selects the next experiment. Algorithmic performance must be reported on donor- and batch-held-out data; otherwise, the model may learn device, material or laboratory signatures instead of human transport biology.

### 6.4. Translational Qualification and Remaining Evidence Gaps

A translational BBB model should be qualified in stages. First, the intended use and decision threshold must be fixed. Second, critical material attributes—composition, ligand density, modulus, swelling, degradation, interface thickness, sorption and extractables—and critical process parameters—mixing, curing, bonding, sterilization, flow and cell seeding—must be linked to acceptance limits. Third, analytical validity requires reference compounds, compound recovery, acellular controls and time-resolved TEER or permeability. Fourth, biological validity requires endothelial identity, junctions, transporters and relevant multicellular responses. Finally, external validity requires blinded interlaboratory studies and prospective comparison with human pharmacokinetic, biomarker or treatment-response data. Table 10 summarizes what the principal application areas have achieved and what still blocks clinical use [12,15,16,17].

The most credible near-term route is therefore not a universal patient-on-a-chip, but fit-for-purpose qualification. Defined matrices and scalable low-sorption devices are appropriate for early transport ranking; patient-derived iPSC or tumor models are justified when genotype- or treatment-specific heterogeneity is the question; and complex printed neurovascular systems are most valuable for mechanisms that require spatial organization. The following section isolates integrated multi-process bioprinting as the manufacturing layer that coordinates separately qualified material modules, while retaining the clinical qualification criteria established here.

## 7. Integrated Multi-Process Bioprinting for Organ-on-a-Chip Manufacturing

**Figure 8:**
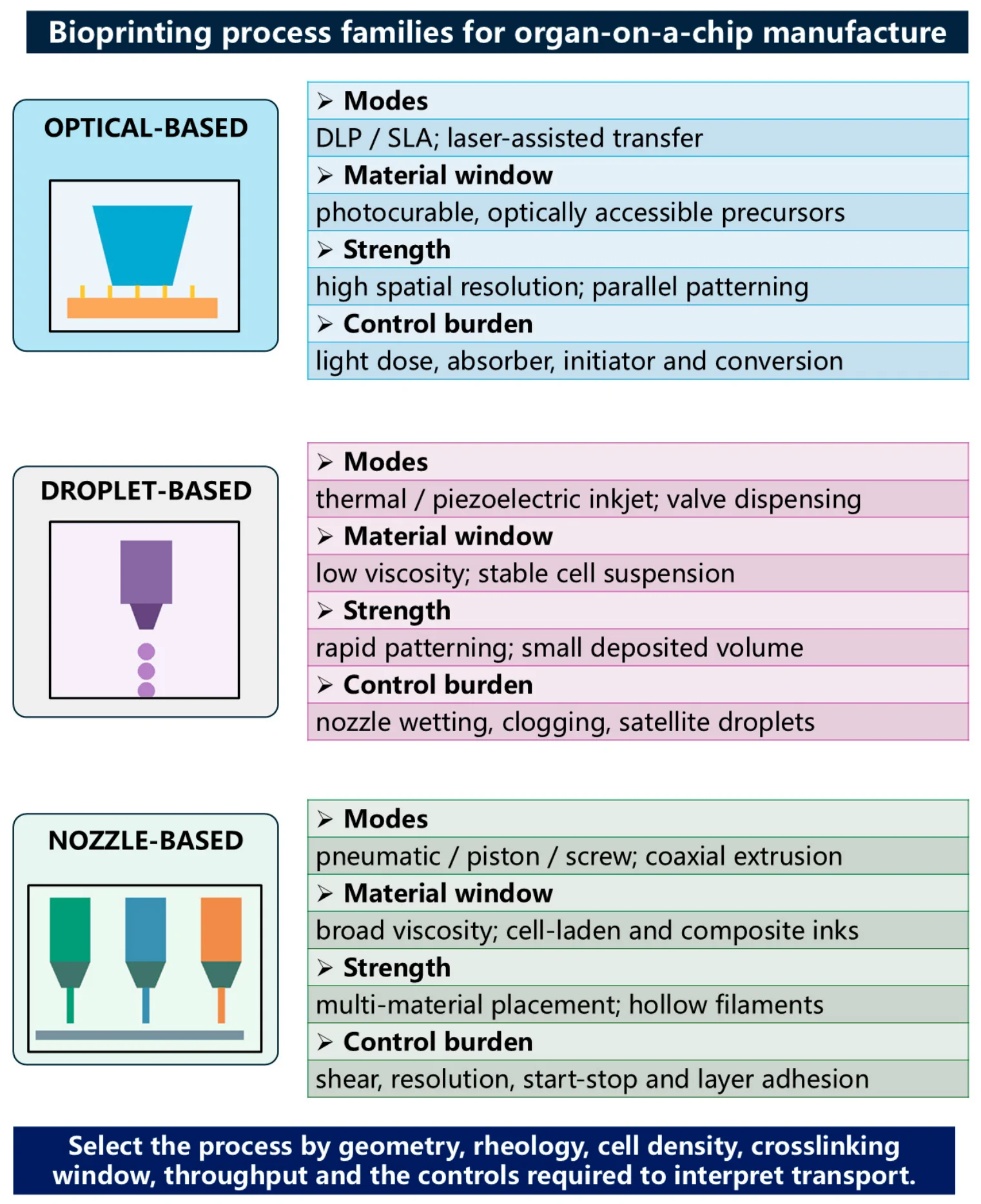
Material-centered taxonomy of optical-, droplet- and nozzle-based bioprinting processes for organ-on-a-chip manufacture. Created by the authors based on technology classifications reported in [104,186,187]; no third-party graphical elements were reproduced.

Section 4.4 evaluated extrusion, coaxial and light-based printing as BBB-specific fabrication routes. The present section addresses a different scale of integration: a multi-process manufacturing cell in which several printheads, dispensing modes and curing steps build an organ-on-a-chip device without repeatedly transferring the construct between instruments [113,117,159,160,161]. Figure 8 is retained only as the process taxonomy from which those modules are selected; the emphasis here is their coordinated use rather than a second comparison of printing principles [104,186,187].

**Figure 9:**
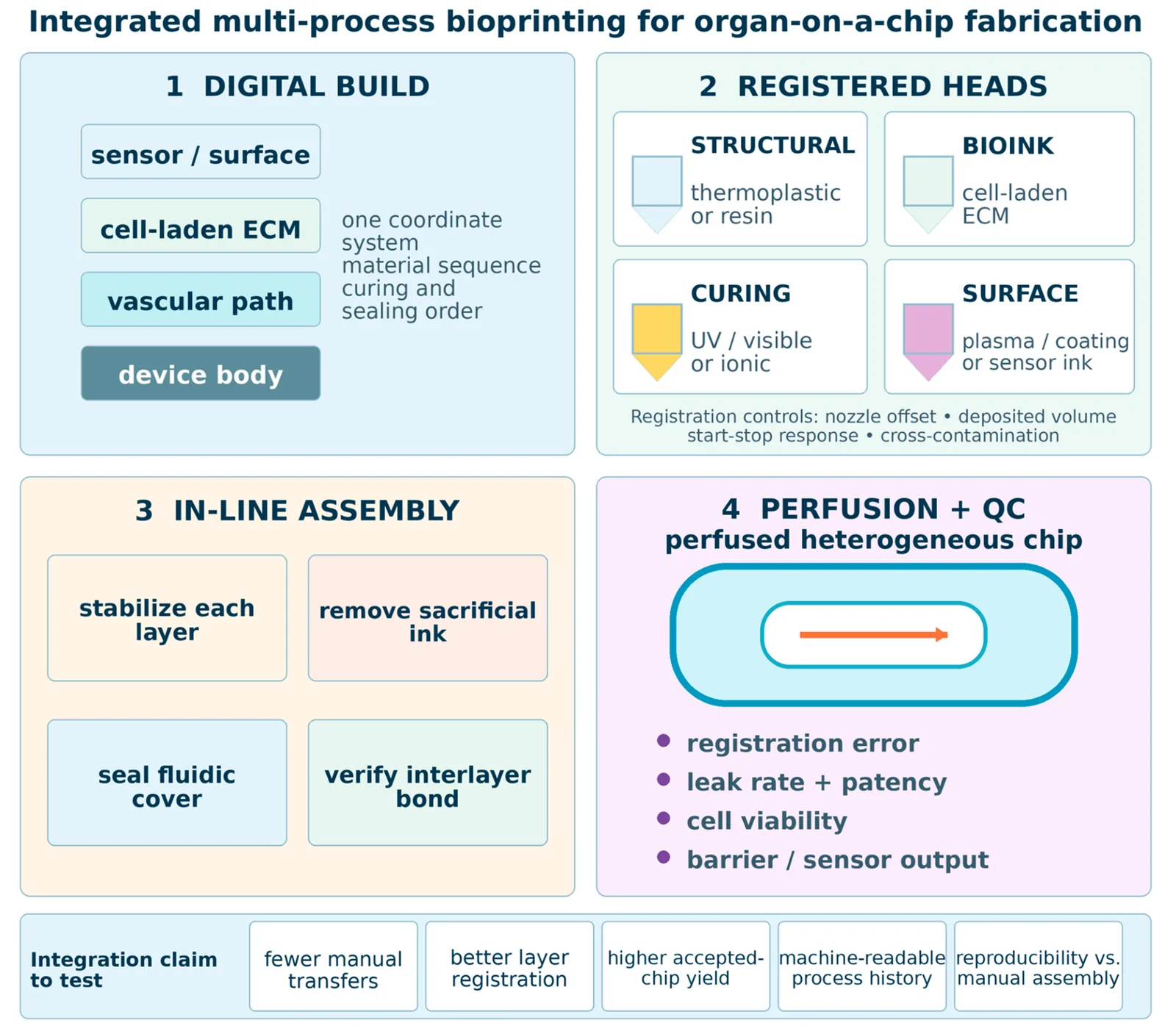
Workflow for integrated multi-process bioprinting of organ-on-a-chip systems, showing a common digital build, registered structural, bioink, curing and surface-processing heads, in-line assembly, perfusion and quality control. Created by the authors based on the integrated fabrication workflows reported by Hamid et al. [160] and Lee and Cho [161]; no third-party graphical elements were reproduced.

An integrated line may combine a thermoplastic or photocurable chip body, a sacrificial vascular template, multiple cell-laden ECM inks, surface-patterning reagents and an electrode or sensor ink. Multi-nozzle deposition assigns these materials to registered coordinates, while in-line ionic, thermal or photochemical stabilization fixes each layer before the next material is introduced [156,157]. Figure 9 illustrates this sequence: digital design and toolpath planning are followed by synchronized structural printing, cell/material deposition, curing or sacrificial-ink removal, sealing and perfusion. Hamid et al. demonstrated the integration concept with four dedicated heads for photopolymer deposition, UV curing, plasma treatment and cell-laden bioink extrusion, whereas Lee et al. reported one-step fabrication of a spatially heterogeneous organ chip [160,161]. The key advance is therefore process orchestration—spatial registration and timing across unlike materials—not the isolated performance of one bioink [19,159,160,161,188].

Multi-process manufacture can reduce manual transfers, preserve alignment between vascular, parenchymal and sensing compartments and make a digital build file the basis of batch reproduction [19,156,157,158,159,160,161]. These benefits are conditional. Nozzle offsets, volumetric calibration, start–stop transients and material carry-over must be measured for every printhead; otherwise nominally registered layers acquire variable wall thickness or cell density. The sequence must also respect incompatible processing windows: Ca^2+^ used for alginate may diffuse into a neighboring formulation, thermal removal of gelatin can soften another phase, and a light dose selected for a structural resin may injure cells or over-crosslink an adjacent hydrogel. Sterile tool changing, closed fluidic sealing and non-destructive inspection are therefore part of the manufacturing method rather than downstream handling [19,104,188].

The most useful near-term platform is a modular printer with independently calibrated deposition, curing and inspection heads rather than an oversized universal machine [18,19]. A transferable workflow should report printhead identity, registration error, deposited volume, cross-contamination controls, curing order, interlayer bond strength, channel patency and leak rate, together with cell viability and functional readouts after the complete build [31,143]. Cost and throughput should be expressed per accepted chip, because faster deposition has little value if post-print sealing or quality-control failure discards a large fraction of devices [144,145]. Compact hardware, rapid sterile printhead exchange and machine-readable process records remain priorities for scalable organ-on-a-chip manufacture [33,143,156].

Accordingly, Section 7 does not propose another BBB printing modality. It defines the integration layer that can combine the BBB-specific material modules evaluated in Section 4.4 with separately manufactured neural, tumor, hepatic or sensing compartments. The central research question is whether a coordinated multi-process build improves registration, yield and interbatch reproducibility relative to assembling the same modules manually. This distinction prevents organ-on-a-chip integration from being conflated with the material-level comparison of BBB bioprinting and provides a direct transition to the data-driven and regulatory-ready platforms discussed next [104,161,188].

## 8. Future Perspectives: Data-Driven, Integrated and Regulatory-Ready BBB Platforms

The next generation of BBB models should not be defined by biological complexity alone. Its value will depend on whether material composition, fabrication variables and biological outputs can be learned from, monitored continuously, coupled to systemic physiology and validated for a stated decision. Six converging directions—artificial-intelligence-guided biomaterials, machine-learning control of BBB chips, integrated biosensors, multi-organ coupling, vascularized brain organoids and regulatory qualification—could turn current prototypes into predictive platforms. At present, however, the findings are uneven: several enabling workflows have been demonstrated outside BBB research, whereas prospective, multisite BBB validation remains scarce [184,185]. Table 11 therefore frames each trend as a testable research program rather than an assumption that added technology will automatically improve prediction.

### 8.1. Artificial-Intelligence-Guided Biomaterial Design

Artificial intelligence can reduce the experimental burden of searching a high-dimensional material space, but only when formulations and outcomes are represented consistently. A BBB-focused dataset should encode polymer identity and molecular weight, functionalization, concentration, crosslinker stoichiometry, curing dose, ligand density, modulus, mesh size, swelling, degradation, protein adsorption and drug sorption together with TEER, permeability, transporter function, junction continuity and cell viability. Seifermann et al. combined a library of more than 900 photodegradable hydrogel microarrays with machine learning and Bayesian optimization, and Xu et al. used iterative Bayesian optimization to relate seven formulation variables to multiple properties of an alginate/polyacrylamide hydrogel [189,190]. These studies demonstrate the workflow, not a ready-made BBB material: neither optimized a human neurovascular phenotype, and their objective functions did not include compound recovery or barrier transport.

The immediate opportunity is therefore multi-objective, uncertainty-aware design rather than a single predicted “best” hydrogel. Active learning could propose the next PEG, GelMA, hyaluronic-acid or composite formulation while constraining cytocompatibility, manufacturability and low drug binding. Physics-informed descriptors and mechanistic priors should prevent chemically implausible recommendations, whereas negative and failed formulations must be retained to avoid survivorship bias. A convincing advance would require prospective fabrication of algorithm-selected materials, comparison with expert-selected controls, and external validation across polymer and cell lots. Without standardized metadata and open raw data, an apparently accurate model may merely reproduce laboratory-specific synthesis practices [184,190].

### 8.2. Machine Learning for BBB-on-a-Chip Optimization and Control

Machine learning can also optimize the complete BBB-on-a-chip process. Candidate inputs include channel and membrane geometry, hydrogel thickness, seeding ratio, flow and oxygen history, maturation time and inflammatory challenge; outputs can combine electrical, optical, biochemical and transport trajectories. In a closed loop, Bayesian optimization or active learning would select a feasible experiment, automated manufacture and sensing would return new measurements, and the model would update its uncertainty. Existing studies have shown explainable molecular prediction of passive BBB penetration and AI-assisted organ-on-a-chip workflows, but BBB-specific closed-loop optimization remains largely prospective [184,185]. The hydrogel studies above establish that iterative materials optimization is technically feasible, while also showing that a BBB objective function must be defined more rigorously [190].

Validation must be deliberately resistant to data leakage. Donors, differentiation runs, material lots and device batches—not individual images or time points from the same chip—should define held-out test sets. Multi-objective models should preserve hard constraints for viability, lumen patency, compound recovery and acceptable permeability rather than maximize TEER alone. Hybrid digital twins that couple mechanistic mass transport and fluid dynamics to data-driven residual models are preferable to opaque predictors because they can distinguish altered barrier biology from adsorption, evaporation or flow failure. Model uncertainty, failed runs and prospective performance in an external laboratory should be reported alongside conventional accuracy [185].

### 8.3. Integrated Biosensors for Real-Time Monitoring

Integrated sensing can convert a terminal assay into a time-resolved experiment. Palma-Florez et al. positioned microscale Cr/Au electrodes near a fibrin-based, multicellular BBB interface and combined micro-TEER with microscopy during permeability evaluation, illustrating how electrical monitoring can be built into the device rather than added after culture [191]. Future systems should combine impedance with selected oxygen, pH, glucose, lactate, cytokine or transporter sensors and optical analysis of junction morphology. Such multimodal measurements could reveal whether a permeability change follows hypoxia, inflammation, metabolic stress or physical leakage, which a single endpoint cannot resolve [185,189].

Sensor integration is itself a biomaterials problem. Electrode, passivation and antifouling layers must withstand sterilization and flow without releasing toxic species, adsorbing the test drug or mechanically disturbing the barrier. “Real-time” should be supported by specified sampling frequency, response time and calibration rather than used as a synonym for repeated measurement [189]. Drift, biofouling, temperature sensitivity and electrode polarization require cell-free controls, in situ reference standards and post-experiment recalibration. The most useful near-term design is a modular, replaceable sensor layer whose analytical performance and biological non-interference are qualified independently before it is used for feedback control [191].

### 8.4. Multi-Organ Chips and Systemic Pharmacology

Multi-organ platforms can address questions that an isolated BBB cannot, particularly when hepatic metabolism, intestinal absorption, renal clearance or immune activation changes the species that reaches the brain. Koenig et al. coupled stem-cell-derived brain, BBB and liver compartments in a closed microphysiological system and tracked parent drugs and metabolites, demonstrating a practical route toward donor-matched neuropharmacokinetics [192]. For CNS discovery, the most informative configurations are likely to be fit-for-purpose BBB–liver or gut–liver–BBB systems rather than indiscriminately large “body-on-a-chip” assemblies.

The key barriers are engineering rather than anatomical. A common medium may not support every tissue; organ-size ratios, fluid volumes, residence times and recirculation can distort exposure; and tubing, bubbles and polymer sorption can destroy mass balance. Future platforms should use low-binding materials, independently matured modules, controlled vascular connections and physiologically based pharmacokinetic models to set flow and scaling. Parent compound, metabolites and total recovery should be measured in every compartment [190]. A multi-organ chip is superior only if coupling explains a clinically relevant exposure or toxicity that its single-organ controls cannot predict [192].

### 8.5. Vascularized Brain Organoids and BBB Assembloids

Vascularized brain organoids offer a complementary path to spatially organized human neurovascular tissue. Sun et al. fused vessel and brain organoids to generate vessel-like networks, BBB-associated structures and microglial interactions, whereas Dao et al. assembled human pluripotent-stem-cell-derived brain and blood-vessel organoids and reproduced molecular and functional features relevant to cerebral cavernous malformations [193,194]. These studies move beyond adding endothelial cells to the organoid surface by allowing vascular and neural programs to interact during assembly.

Their future utility depends on material and perfusion control. Chemically defined matrices should replace variable basement-membrane extracts; adhesive, degradable and angiogenic cues should be patterned to guide vascular invasion; and perfusable lumens should provide controlled shear and luminal delivery [193]. Major gaps include immature or fetal-like phenotypes, organoid-size and network variability, diffusion-limited cores, incomplete vessel perfusion and the absence of a controlled luminal-to-abluminal transport path. Consequently, vascular markers or vessel-like morphology alone are insufficient. Defined reference compounds, quantitative permeability, compound recovery and comparison with a microfluidic BBB standard are needed before these systems can support drug ranking [194].

### 8.6. Standardized Validation for Regulatory Use

Regulatory progress requires a context of use, not a claim that one BBB model can replace every animal experiment. OECD good in vitro method practices provide a quality framework for personnel, apparatus, test systems, controls, reporting and data integrity, while the 2025 FDA roadmap signals increasing support for qualified new approach methodologies. Neither document constitutes automatic acceptance of a BBB chip [195]. The IQ MPS workshop likewise emphasized that complex in vitro models require broadly applicable standards plus context-specific qualification rather than one universal protocol [195,196].

A practical BBB validation dossier should contain three layers. Technical qualification should specify critical material attributes and process parameters, dimensional tolerances, extractables, sorption, leakage, sensor calibration and lot release. Organ-specific qualification should use prespecified reference compounds spanning passive diffusion, efflux and receptor-mediated transport, with TEER or impedance, tracer permeability, junctions, transporters, viability and mass balance [47,105]. Context-specific qualification should define the decision threshold, blinded performance, interlaboratory reproducibility and prospective concordance with human pharmacokinetic or clinical data [195]. Raw data, exclusions, protocol deviations and versioned analysis code should be auditable. Existing meta-analysis and chip reviews show that inconsistent cells, materials, geometry and endpoints remain the central obstacle to such qualification [196,197]. Research roadmap for future BBB biomaterials and manufacturing platforms, are summarized in Table 11.

The priorities can now be ordered. In the near term, shared metadata, material release criteria, sensor calibration, reference-compound panels and interlaboratory studies are more urgent than adding further compartments [184,185]. The next stage is prospective closed-loop optimization and PBPK-informed multi-organ coupling using low-sorption, manufacturable devices. Vascularized organoids should enter decision workflows only after defined matrices, controlled perfusion and quantitative transport are established [189,191]. Across all three horizons, the decisive question is not whether a model appears more human, but whether a controlled material and manufacturing change yields a reproducible, interpretable and clinically concordant decision [193,194]. This findings-driven sequence would allow artificial intelligence and biological complexity to amplify validated measurements rather than amplify uncontrolled variation [189,196,197].

## 9. Conclusions and Outlook

This review identifies material chemistry and processing as central determinants of in vitro BBB and NVU performance. Matrix ligands, mechanics, degradability, interface thickness, flow and device-body sorption act together, so no platform is universally optimal. Standardized Transwell systems remain useful for controlled screening, whereas hydrogel micropillar, tubular, self-assembled and printed systems offer more direct cell–matrix interaction or vascular geometry at the cost of additional fabrication variables [15,41,44,128,146]. Natural matrices provide bioactivity but vary and contract; defined PEG-based networks improve reproducibility but require programmed adhesion and degradation; GelMA, HAMA and composite systems balance these properties while introducing crosslinking and processing controls [12,24,41,47,70,106].

The most informative recent studies change a defined material feature—peptide identity, basement-membrane thickness, photocrosslinking, fiber guidance or coaxial organization—and relate it to barrier or vascular outcomes [42,46,70]. Direct comparison nevertheless remains limited by inconsistent cells, geometry, shear and readouts. Future work should use matched experimental conditions, acellular transport controls and combined TEER, tracer, junctional and transporter analyses, while reporting precursor source, functionalization, crosslinking, modulus, swelling, degradation and compound recovery [12,15]. Defined matrices, low-sorption scalable devices, integrated sensing and validated long-term culture are more likely to improve predictive value than added complexity alone.

To make this transition, studies should separate material effects from biological variability through factorial designs with independently controlled chemistry, mechanics, ligand density, geometry and flow. Each experiment should report batches, acellular transport, compound mass balance, viability, junction continuity and lumen patency. Interlaboratory work should then test whether the same formulation and fabrication protocol reproduce barrier function across sites. Open reporting of synthesis, curing and raw permeability data would support meaningful comparison and meta-analysis [12,16]. These practices will benchmark advanced systems against simpler controls and identify genuine benefits of added complexity.

## Figures and Tables

**Table 1:** Location-to-material map of the principal BBB/NVU cells and molecules discussed in Section 2.

Cell/Molecule	Native Localization	Biomaterial Variable	Representative Readout	Primary Functions in BBB/NVU	Representative Reference
Brain microvascular endothelial cells	Luminal vascular wall	Surface ligand density, shear, stiffness and drug sorption	TEER; ZO-1/CLDN5/OCLN; efflux; tracer permeability	Engineered basement membrane improved iPSC-derived barrier properties.	[28,30,42]
Collagen IV, laminin, fibronectin and proteoglycans	Thin abluminal endothelial–pericyte interface	Interface thickness, modulus, porosity and ligand presentation	Adhesion; polarity; deposited basement membrane; permeability	Engineered basement membrane and lyophilized dECM film changed barrier performance.	[42,43]
Pericytes	Embedded in or directly adjacent to basement membrane	Endothelial–pericyte distance and matrix degradability	Coverage; PDGFRβ/NG2; vessel stability; permeability	Fibrin and collagen tri-cultures enabled direct multicellular contact.	[44,45]
Astrocyte endfeet	Outer abluminal vascular surface	Softness, topology and integrin-binding motifs	GFAP; AQP4 polarization; capillary-network yield	Collagen I microfibers promoted astrocyte survival and capillary maturation.	[46]
Tight-junction proteins, transporters and glycocalyx	Endothelial borders and polarized luminal/abluminal surfaces	Mesh permeability, flow conditioning and low-fouling surface chemistry	TEER; tracer size series; P-gp; junction continuity	GelMA, collagen and engineered interfaces produced different barrier readouts.	[42,45,47]
Microglia	Perivascular and parenchymal compartments	Degradability and inflammation-responsive chemistry	Iba1 morphology; cytokines; barrier loss/recovery	Best included only for immune-remodeling questions; matrix-only controls remain necessary.	[12,48]
Neurons	Parenchyma close to microvessels	Soft, permissive or electroactive matrix and controlled separation	Neurites; synaptic markers; metabolic coupling	Linked NVU chips resolved endothelial–neuronal metabolic coupling.	[9]

**Table 2:** Representative hydrogel materials, incorporated ligands or peptides, preparation methods, BBB/NVU applications and limitations.

Preparation or Crosslinking	Peptide/Ligand	Application and Reported Outcome	Advantages	Limitations	Representative Reference
Covalent PEG network; ligand conjugation	RGD, IKVAV and degradable sequences	Endothelial–astrocyte 3D BBB co-culture; improved biomimetic cell–matrix signaling	Defined chemistry; independently tunable	Bioinert without functionalization; diffusion depends on mesh size	Peptide–PEG [70]
0.05% thiol-HA, 1.5% GelMA, 15% ionic polysaccharide, 0.1% I2959; 365 nm light for 2 s plus ionic maturation	IKVAV plus gelatin-derived motifs	Approximately 300 Pa; stable perfusable lumen, junctions and low small-molecule permeability for at least 10 days	Brain-soft, nonfibrillar matrix; specified ligand and hybrid crosslinking	Commercial component; mixed crosslinking complicates attribution; UV/initiator and PDMS controls required	Thiol-HA/GelMA/VitroGel [107]
0.05% thiol-HA, 1.5% GelMA and 0.1% I2959; 365 nm photocrosslinking	Gelatin-derived motifs; no added peptide	Supported astrocyte culture but was too soft to retain a cylindrical BBB lumen and showed weak endothelial attachment	Simple, brain-ECM-related HA formulation	Channel collapse; inadequate endothelial adhesion without a secondary ligand-bearing phase	Thiol-HA/GelMA base matrix [107]
Methacrylation plus photoinitiated crosslinking	Gelatin-derived integrin motifs	BBB model for breast-cancer brain metastasis	Patternable; stiffness tunable by dose	Light/initiator cytotoxicity; batch-dependent gelatin	GelMA [47]
Neutralization and thermal fibrillogenesis	Native collagen-binding sites	Stem-cell-derived human BBB hydrogel model	Simple, cell-remodelable 3D matrix	Weak mechanics; contraction; lot variability	Collagen I hydrogel [45]
Microfiber fabrication and incorporation in 3D MPS	Native collagen motifs plus contact guidance	Promoted brain capillary-network formation	Adds anisotropy and vascular guidance	Fiber geometry and handling require control	Collagen I microfibers [46]
Thrombin-mediated fibrin polymerization	Native integrin-binding sites	Self-organized endothelial–pericyte–astrocyte microvasculature	Supports angiogenesis and multicellular contact	Rapid remodeling; variable compaction	Fibrin self-assembly [44]
PEG-maleimide thiol coupling	Protease-cleavable crosslinks	On-demand remodeling/release in neural interfaces	Programmable degradation	Not originally validated as a complete BBB model	MMP-sensitive PEG [96]
Collagen I/fibrin/HA/PEGDA combined crosslinking	Native ECM motifs	CNS matrix modeling under elevated fibrin	Broader mechanical/biochemical range	Complex kinetics; attribution is difficult	Quad-network composite [102]
Preformed phase-separated porous microgels	Matrix-associated adhesion sites	NPC delivery and vascularization after stroke	Injectable; dual porosity	Evidence of in vivo repair, not direct BBB validation	Injectable microgels [97]
RAFT-synthesized PNIPAM grafted to cellulose nanocrystals; thermal gelation	No added peptide; cellulose/PNIPAM interface	Brain-cell-compatible injectable scaffold with paclitaxel release	Sol at room temperature; rapid gelation near body temperature	Brain implant/scaffold, not a complete perfused BBB model	CNC–g–PNIPAM [86]

**Table 3:** Representative stimuli-responsive hydrogel studies published within the past five years, with preparation routes, material responses and BBB-specific findings.

Trigger and Recognition Chemistry	Preparation Route	Reported Response or Result	BBB Relevance and Principal Limitation	Representative Reference
Temperature; PNIPAM dehydration and LCST transition	Controlled PNIPAM synthesis followed by grafting to cellulose nanocrystals	Injectable gelation near body temperature; compatibility with brain endothelial cells and astrocytes; paclitaxel release	Brain-implant records, but no perfused lumen, TEER or BBB permeability validation	CNC–g–PNIPAM [86]
Temperature; LCST-driven volume-phase transition	NIPAAm, chitosan and 0.1–3% CNC with I2959; nitrogen purge and 30-min photopolymerization	Transition at 34–38 °C; storage modulus increased above 30 °C	Useful synthesis/property benchmark; no BBB cells or barrier endpoints	PNIPAM/chitosan/CNC [108]
MMP-2 cleavage of PLGLAG peptide	Self-assembly of peptide-amphiphile nanofibers bearing cleavable linker and QK peptide	Enzyme-dependent QK release; endothelial migration/tube formation; improved junction markers and leakage in cerebral ischemia/reperfusion	Direct BBB-related in vivo findings, but not an in vitro BBB material model	PA–TIMP–QK [109]
UV-induced [2+2] photodimerization of supramolecular junctions	HA functionalization, CB8 host–guest gelation and patterned illumination	Local conversion to covalent crosslinks; spatially tuned viscoelasticity and vessel-like tube formation	Demonstrates post-gel phototuning; lymphatic rather than BBB validation	HA–merocyanine/CB8 [110]
405 nm photocleavage of linker strands	Y-motif DNA and linker assembly in water-in-oil droplets	Selected bead disassembly and localized morphogen release in retinal organoids	Organoid-scale spatial control; dose, diffusion and BBB-cell compatibility remain untested	DNA hydrogel microbeads [111]
Ultrasound; piezoelectric and electrokinetic conversion	KNN nanocrystals and rGO dispersed in a gelatin/PVA composite network	Ultrasound-enhanced electrokinetic and angiogenic effects	Neurovascular wound model, not a BBB model; filler retention and heating require control	gelatin/PVA–KNN–rGO [99]

**Table 4:** Representative Transwell and membrane-supported BBB studies, with their biomaterial variables and reported consequences.

Load-Bearing Interface	ECM Material and Preparation	Reported Result	Biomaterial Interpretation and Limitation	Representative Reference
12 mm collagen-coated insert; 0.4 versus 3.0 μm pores	Basolateral poly-L-lysine; fibronectin used for endothelial expansion	3.0 μm pores and closer astrocyte/pericyte contact produced higher, more stable TEER	Pore size and coating change cell contact; large pores may permit migration	[127]
Conventional Transwell membrane	Endothelial cells and astrocytes mixed with thermally gelling Matrigel	Simplified 3D model assembled in approximately 30 min	Rapid and bioactive, but undefined tumor-derived matrix and lot variability	[126]
Standard Transwell incorporated into millifluidic housing	Protein-coated insert supporting endothelial–astrocyte–pericyte culture	Added physiological flow while retaining TEER/permeability-compatible insert workflow	Separates membrane chemistry from shear, but retains a rigid planar separator	[125]
0.4 μm insert supporting a 3 mm collagen I gel	5 mg/mL collagen I with optional astrocytes; collagen IV/fibronectin surface coating	TEER > 700 Ω·cm^2^; low paracellular transport and functional transporter assays	Defined preparation and 3D glial matrix; collagen contraction and diffusion lag require controls	[45]
Sparse electrospun nanofiber support	Ultrathin ECM-hydrogel engineered basement membrane	Approximately 500 kPa interface; high physical barrier and efflux-pump activity	Closer control of thickness, stiffness and ECM chemistry; more complex fabrication	[42]

**Table 6:** Recent 3D-printed BBB and neurovascular systems, emphasizing bioink chemistry and material–process trade-offs.

Printing route/Geometry	Material System and Stabilization	Cell–Material Organization	Reported Material-Related Outcome	Limitation Requiring Control	Representative Reference
DLP; branching perfusable lumen network	PEGDA + 15% GelMA; light curing; collagen I/Heprasil/Matrigel evaluated for adhesion	Astrocytes in printed bulk; endothelial cells and pericytes introduced into lumens	PEGDA supplied print stability; GelMA and ECM additives improved attachment and imaging compatibility	Printed lumens remained larger than capillaries; stiffness, coating and cell density were changed together	[162]
DLP; perfusable channels with tunable topology	PEG-NB thiol–ene network; spatial RGD, IKVAV and HAVDI functionalization	Endothelium on peptide-defined walls; astrocytes in RGD-functionalized bulk	HAVDI/IKVAV improved endothelial coverage and junctional ZO-1; RGD supported co-culture	Capillary-scale resolution and matched peptide/mechanics controls remain necessary	[95]
Extrusion bioprinting; neurovascular tissue construct	Low-viscosity collagen composite reinforced with alginate; thermal/ionic stabilization	Neural and vascular cells distributed through a printable ECM-like composite	Alginate improved printability while collagen supplied remodeling and adhesion cues	Alginate fraction and Ca^2+^ alter stiffness, porosity and adhesion; direct BBB transport validation is needed	[164]
Projection stereolithography; perfusable 700 μm channel	10 wt% GelMA + 3.25 wt% PEGDA; LAP, tartrazine and glycerol; light curing	Astrocytes embedded; pericytes and endothelial cells seeded in the lumen	Hybrid ink combined adhesion, viscosity, optical control and structural stability for perfusion and screening	Photochemistry, leachables, swelling and channel scale may confound barrier transport	[163]
Coaxial extrusion; three concentric vascular layers	Cell-laden hydrogel streams with rapid core–shell stabilization	Endothelial, pericyte and astrocyte layers placed radially	Multilayer placement supported a BBB-like construct and drug-screening workflow	Flow-rate mismatch, interlayer mixing and crosslink gradients change wall thickness and permeability	[24]

**Table 7:** Material-centered comparison of representative BBB/NVU studies.

Material Strategy	Preparation/Design Variable	Reported Contribution	Unresolved Limitation	Representative Reference
Peptide-functionalized PEG	Covalent network and ligand incorporation	Defined 3D endothelial–astrocyte matrix	Requires validation across stiffness/ligand combinations	[70]
Peptide-patterned PEG-NB	DLP topology plus spatial RGD/IKVAV/HAVDI	Separated wall and bulk biochemical cues	Printing, peptide density and mechanics remain coupled	[95]
Fibrin self-assembled network	Cell-driven vasculogenesis	Perfusable multicellular microvessels	Network geometry and yield vary	[44]
GelMA hydrogel	Methacrylation and photocrosslinking	Disease-oriented BBB model	Light/initiator and gelatin variability	[47]
Collagen hydrogel	Thermal fibrillogenesis	Human stem-cell-derived BBB environment	Contraction and fibril heterogeneity	[45]
Collagen I microfibers	Topographic reinforcement	Promoted brain capillary-network formation	Fiber dimensions and placement need standardization	[46]
Engineered basement membrane	Controlled vascular support interface	Enhanced iPSC-derived barrier properties	Long-term and interlaboratory validation needed	[42]
IKVAV-functionalized HA/gelatin	Tumor–matrix–vascular interface in a microfluidic model	Resolved glioblastoma effects on permeability and adhesion	Matrix and tumor-state effects require matched controls	[107]
PEGDA/GelMA printed scaffold	DLP, perfusable lumen and ECM coating	Multicellular human brain microvasculature	Lumen scale, coating and stiffness changed together	[162]
GelMA/PEGDA printed scaffold	Projection stereolithography and photoabsorber-controlled curing	Perfusable scalable BBB screening format	Photochemistry, swelling and extractables require controls	[163]

**Table 8:** Preparation and synthesis routes for material classes used in BBB and NVU models.

Material Class	Preparation or Synthesis Example	Introduction/Stabilization in the Model	Variables That Must Be Controlled	Representative Reference
Collagen I	Dilute acidic stock on ice; neutralize with base and buffered salts; mix with cells	Inject or pin in a compartment and warm to 37 °C for fibrillogenesis	Source, concentration, pH, ionic strength, temperature, fibril size and contraction	[45,139,141]
Fibrin	Dissolve fibrinogen; combine with thrombin and Ca2+, with cells added before gelation	Cast in a chamber or micropillar lane; enzymatic polymerization occurs in situ	Fibrinogen/thrombin dose, gelation time, compaction, degradation and lot variability	[44,131]
Matrigel/basement-membrane extract	Thaw and mix on ice to prevent premature gelation; optionally dilute with medium or collagen	Load cold and warm to 37 °C for thermal gelation	Lot composition, protein concentration, gelation temperature, contraction and tumor-derived origin	[11,126]
GelMA and HAMA	React gelatin or HA hydroxyl/amine groups with methacrylic anhydride; dialyze, lyophilize and redissolve with LAP or another initiator	Cast or print, then expose to defined 365–405 nm light; wash unreacted species	Degree of substitution, initiator, wavelength, irradiance, exposure, conversion and swelling	[47,105,163]
Alginate	Dissolve sodium alginate; optionally conjugate RGD through carbodiimide chemistry	Extrude into CaCl_2_ or use internal Ca^2+^ release; combine with collagen/GelMA for adhesion	G/M ratio, molecular weight, Ca^2+^ dose, crosslink gradient, porosity and ligand density	[24,164]
PEGDA and PEG-NB	Use acrylated or norbornene-functional multi-arm PEG; add dithiols/cysteine peptides and RGD, IKVAV, HAVDI or degradable sequences at known stoichiometry	Photopolymerize by chain growth or thiol–ene reaction; spatially pattern by DLP	Functionality, thiol:ene ratio, peptide density, conversion, initiator/light dose and extractables	[70,95,162]
PVA/polyacrylamide	PVA: repeated freeze–thaw or chemical crosslinking/photocrosslinking; polyacrylamide: radical copolymerization of acrylamide and bisacrylamide	Form defined non- or slowly degradable mechanical substrates, followed by ligand coupling where needed	Residual monomer/crosslinker, washing, modulus, nonadhesive surface and long-term stability	[104]
Decellularized ECM	Detergent/enzyme decellularization; extensive washing; DNA/residual-detergent testing; lyophilization, milling and pepsin digestion	Neutralize the digest and thermally gel, or blend into a printable carrier	Donor/tissue source, retained ECM, DNA, detergent, batch mechanics and gelation	[41,106]
Composite/IPN/fiber-reinforced gels	Mix two compatible precursors before curing, crosslink networks sequentially, or disperse fibers/microgels before primary gelation	Use orthogonal thermal, enzymatic, ionic or photo steps to avoid premature phase separation	Order of formation, interfacial bonding, phase separation, anisotropy, swelling and diffusion	[46,164]
Sacrificial and support inks	Prepare gelatin or Pluronic F127 at temperature-dependent printable concentration	Print a template inside a structural gel; liquefy or dissolve after surrounding-network stabilization	Removal completeness, osmotic/thermal exposure, residue, channel collapse and lumen roughness	[22,25]
PDMS	Mix elastomer base and curing agent, commonly 10:1; degas, cast on an SU-8 master and thermally cure	Punch ports and oxygen-plasma bond to glass or another PDMS layer; coat the channel with ECM protein	Oligomers, hydrophobic-drug sorption, plasma aging, gas permeability, bond strength and leakage	[147,148,149]
Glass, PMMA, COC/COP and porous PC/PET/PTFE	Glass: etch or micromachine; thermoplastics: hot emboss or injection mold; membranes: track-etch or fabricate porous films and hydrophilize as needed	Thermal, solvent, adhesive or plasma-assisted bonding; laminate membranes between channels	Bonding residue, deformation, optical quality, surface activation, pore size/thickness and compound recovery	[11,15,16,17,135,138]

**Table 9:** Decision-oriented comparison of BBB/NVU fabrication platforms for research, pharmaceutical screening and scale-up.

Platform/Material System	Reproducibility	Scale + HTS	Relative Cost	Long-Term Stability	Standardization	Preferred vs. Limiting Trade-Offs	Representative Reference
Transwell or membrane insert	High	High	Low	Medium–high	High	Preferred for routine TEER/permeability and compound ranking; planar stiff membrane, no physiological lumen and limited shear.	[42,45,125,126,127,128]
Cast defined hydrogel (PEG/GelMA)	Medium–high	High	Low–medium	Medium–high	Medium–high	Preferred for factorial chemistry/mechanics screens; requires ligand and curing controls and usually lacks controlled perfusion.	[47,70,95]
PDMS microfluidic chip	Medium	Low–medium	Low prototype/high labor	Medium	Low–medium	Preferred for rapid prototyping, flow and live imaging; drug sorption, oligomers, bubbles and bonding complicate pharmaceutical use.	[9,15,16,17,133,134,135,136,137,138,146,147,148,149]
Glass or thermoplastic chip	High after tooling	High after tooling	High tooling/low unit	High	Medium–high	Preferred for compound recovery, automation and industrial batches; slower design iteration and more demanding bonding/surface activation.	[15,16,17]
Hydrogel-micropillar chip	Medium	Medium	Medium	Medium	Medium–low	Preferred for direct endothelial–ECM contact and imaging; gel leakage, contraction and pinning depend on viscosity and gelation kinetics.	[11,107,146]
Templated tubular lumen	Medium	Low–medium	Medium	Medium	Low–medium	Preferred for circumferential endothelium and flow–transport studies; template alignment, TEER access and abluminal sampling limit throughput.	[139,141]
Self-assembled microvascular network	Low–medium	Low–medium	Medium–high	Low–medium	Low	Preferred for capillary morphogenesis, remodeling and invasion; network yield, connectivity and fibrin compaction vary between batches.	[44,46,131]
Spheroid or organoid	Low–medium	Medium–high	Medium	Medium	Low–medium	Preferred for multicellular disease phenotypes and multiwell imaging; lacks controlled perfusion and has size, necrosis and batch variability.	[12,119,120,121,122,123,124]
DLP/SLA bioprinting	High geometry/medium material	Medium–high	High	Medium–high	Medium	Preferred for reproducible channels and spatial ligand patterning; photochemistry, swelling, resolution and non-capillary dimensions require controls.	[95,162,163]
Extrusion/coaxial bioprinting	Medium	Medium	Medium–high	Medium	Low–medium	Preferred for multilayer, high-cell-density and multi-material placement; nozzle shear, resolution and interlayer mixing reduce consistency.	[24,164]

**Table 10:** Clinical applications enabled by biomaterials and advanced BBB-model manufacturing, with current translational findings and limitations.

Clinical Application/Findings	Biomaterial and Manufacturing Innovation	Translational Contribution	Principal Unresolved Limitation	Representative Reference
Patient-specific BBB phenotyping	Patient-derived iPSC lineages in an ECM-coated perfused organ-on-a-chip	Links donor genotype to barrier and transporter phenotypes; enables isogenic or patient-group comparisons	Clone-to-clone differentiation, endothelial identity, long timelines and undefined matrix components	[176]
CNS delivery and antibody shuttling	Hypoxia-conditioned iPSC endothelium, ECM coating, pericyte/astrocyte support and flow	Sustained barrier plus efflux and receptor-mediated transport for ranking delivery strategies	PDMS sorption, limited throughput and incomplete concordance with human exposure	[177]
Parkinson’s disease	Perfused multicellular chips and patient-derived astrocytes embedded beside endothelial microvessels	Connects alpha-synuclein or LRRK2-associated inflammation to BBB failure and target rescue	Donor and network variability; mechanisms have not been prospectively linked to treatment response	[178,179]
Alzheimer’s disease	Communicating vascular and neural hydrogels supporting perfusion and at least four weeks of culture	Captures progressive permeability, vascular dysfunction and amyloid-beta distribution	Mixed donor sources and coupled matrix variables limit patient-specific causal attribution	[180]
Glioblastoma therapy selection	Patient-derived cells, brain dECM bioink, spatial printing and modular BBB/tumor assembly	Models drug access together with tumor response and can accommodate different tissue-maturation schedules	Sample heterogeneity, dECM batches, turnaround time and insufficient prospective clinical cohorts	[182,183]
AI-assisted compound prioritization	Standardized plate-compatible interfaces, automated sensing and machine-readable longitudinal outputs	Can integrate transport, toxicity, imaging and omics for active-learning screens	Training labels are heterogeneous; direct BBB-chip/AI validation and external test cohorts remain scarce	[184,185]

**Table 11:** Research roadmap for future BBB biomaterials and manufacturing platforms.

Emerging Direction	Current Findings	Priority Material/Process Variables	Near-Term Deliverable	Critical Gate to Translation	Representative Reference
AI-guided biomaterials	Large hydrogel libraries and Bayesian optimization have been demonstrated outside BBB-specific applications.	Composition, crosslinking, ligand density, mechanics, degradation, swelling and drug sorption.	Open, machine-readable BBB material dataset and prospective Pareto-optimal formulations.	External fabrication across material and cell lots; improvement over expert-selected controls.	[189,190]
ML-controlled BBB chips	Permeability prediction and organ-on-a-chip/AI workflows exist; closed-loop BBB optimization is still limited.	Geometry, flow, oxygen, cell ratio, maturation, compound recovery and multimodal barrier outputs.	Uncertainty-aware active-learning loop with automated manufacture and sensing.	Donor-, batch- and laboratory-held-out validation; interpretable failure detection.	[184,185,189,190]
Integrated biosensors	On-chip micro-TEER with imaging has enabled longitudinal permeability studies.	Electrodes, passivation, antifouling, calibration, sampling rate and biological non-interference.	Modular impedance plus metabolic or biochemical sensor panel.	Drift and fouling control; analytical calibration and proof that sensors do not perturb the BBB.	[191]
Multi-organ platforms	A stem-cell-derived brain–BBB–liver chip has tracked parent drugs and metabolites.	Low-binding circulation path, compatible media, organ scaling, residence time and mass balance.	PBPK-informed BBB–liver or gut–liver–BBB system with single-organ controls.	Reproducible exposure and metabolite prediction superior to uncoupled modules.	[192]
Vascularized organoids	Vessel–brain fusion and BBB assembloids reproduce neurovascular interactions and disease features.	Defined matrices, vascular guidance, perfusable lumens, oxygen and maturation.	Perfused, defined-matrix assembloid with controlled luminal access.	Batch reproducibility, adult-like phenotype and quantitative transport with reference compounds.	[193,194]
Regulatory validation	GIVIMP, regulatory NAM roadmaps and MPS standardization principles provide a framework.	Critical quality attributes, acceptance limits, reference compounds, SOPs and data provenance.	Context-of-use dossier and blinded multisite ring trial.	Interlaboratory precision and prospective concordance with human outcomes.	[47,105,195,196,197]

## Data Availability

No new data were created or analyzed in this study. Data sharing is not applicable to this article.

## Abbreviations

The following abbreviations are used in this manuscript:

BBB	Blood–brain barrier
NVU	Neurovascular unit
dECM	Decellularized extracellular matrix
TEER	Transendothelial electrical resistance
MMP	Matrix metalloproteinase
IPN	Interpenetrating Network
